# PHGDH drives 5-FU chemoresistance in colorectal cancer through the Hedgehog signaling

**DOI:** 10.1186/s13046-025-03447-y

**Published:** 2025-07-10

**Authors:** Caterina Mancini, Giulia Lori, Gianluca Mattei, Marta Iozzo, Dayana Desideri, Fabio Cianchi, Laura Fortuna, Federico Passagnoli, Daniela Massi, Filippo Ugolini, Luca Messerini, Salvatore Piscuoglio, Antonio Pezone, Francesca Magherini, Alessio Biagioni, Tiziano Lottini, Demetra Zambardino, Giuseppina Ivana Truglio, Elena Petricci, Alberto Magi, Annarosa Arcangeli, Luisa Maresca, Barbara Stecca, Erica Pranzini, Maria Letizia Taddei

**Affiliations:** 1 Department of Experimental and Clinical Medicine, University of Florence, Florence, Italy; 2 Department of Health Sciences Clinical Pharmacology and Oncology Unit, University of Florence, Florence, Italy; 3 Department of Experimental and Clinical Biomedical Sciences, University of Florence, Florence, Italy; 4 Department of Health Sciences, University of Florence, Florence, Italy; 5 IRCCS Humanitas Research Hospital, Rozzano, Milano Italy; 6 Department of Biology, University of Naples “Federico II”, Naples, Italy; 7 Department of Biotechnology, Chemistry and Pharmacy, University of Siena, Siena, Italy; 8 Department of Information Engineering, University of Florence, Florence, Italy; 9 Core Research Laboratory, ISPRO, Florence, Italy

**Keywords:** Phosphoglycerate dehydrogenase, Chemo-resistance, 5-Fluorouracil, Colorectal cancer, Serine, Stemness, Hedgehog signaling

## Abstract

**Background:**

Phosphoglycerate dehydrogenase (PHGDH) is the rate-limiting enzyme in the de novo Serine synthesis pathway (SSP), a highly regulated pathway overexpressed in several tumors. Specifically, PHGDH expression is dynamically regulated during different stages of tumor progression, promoting cancer aggressiveness. Previously, we demonstrated that high Serine (Ser) availability, obtained by increased exogenous uptake or increased PHGDH expression, supports 5-Fluorouracil (5-FU) resistance in colorectal cancer (CRC). Beyond its metabolic role in sustaining Ser biosynthesis, different “non-enzymatic roles” for PHGDH have recently been identified. The present study aims to investigate non-enzymatic mechanisms through which PHGDH regulates 5-FU response in CRC.

**Methods:**

Overexpression and gene silencing approaches have been used to modulate PHGDH expression in human CRC cell lines to investigate the role of this enzyme in 5-FU cellular response. Identified mechanisms have been validated in selected 5-FU resistant cell lines, CRC patients-derived tumor tissue samples, and patients-derived 3D organoids. Transcriptomic analysis was performed on wild-type and PHGDH-silenced cell lines, allowing the identification of pathways responsible for PHGDH-mediated 5-FU resistance. The relevance of identified genes was validated in vitro and in vivo in a CRC xenograft model.

**Results:**

PHGDH expression is highly variable among CRC tissues and patient-derived 3D organoids. A retrospective analysis of CRC patients highlighted a correlation between PHGDH expression and therapy response. Coherently, the modulation of PHGDH expression by gene silencing/overexpression affects 5-FU sensitivity in CRC cell lines. Transcriptomic analysis on CRC cell lines stably silenced for PHGDH evidenced down regulation in Hedgehog (HH) pathway. Accordingly, in vitro and in vivo studies demonstrated that the combined treatment of 5-FU and HH pathway inhibitors strongly hinders CRC cell survival and tumor growth in CRC xenograft models.

**Conclusions:**

PHGDH sustains 5-FU resistance in CRC by mediating the upregulation of the HH signaling; targeting the here identified PHGDH-HH axis increases 5-FU susceptibility in different CRC models suggesting the 5-FU/HH-inhibitors combinatorial therapeutic strategy as a valid approach to counteract drug resistance in CRC.

**Supplementary Information:**

The online version contains supplementary material available at 10.1186/s13046-025-03447-y.

## Background

Metabolic reprogramming has emerged as a hallmark of cancer and a crucial determinant of tumor progression. Increasing evidence supports the central role of Serine (Ser) metabolism to sustain the synthesis of proteins, lipids, and nucleotides required for rapid cancer cell proliferation, maintaining the balance of folate metabolism and redox homeostasis [1]. Phosphoglycerate dehydrogenase (PHGDH) is the first rate-limiting enzyme of the Ser synthesis pathway (SSP). PHGDH levels are increased in different types of cancers and correlate with tumor malignancy [2–5]; indeed, many cancer cells are dependent on Ser availability, and PHGDH inhibition impairs tumor cell growth in vitro and in vivo [6, 7]. However, starting from early studies investigating the role of PHGDH in tumorigenesis, it emerged that the supplementation of exogenous Ser, or a cell-permeable methyl-serine-ester, does not abolish detrimental effects of the PHGDH knockdown on tumor cells, arguing the contribution of broader Ser-independent effects for PHGDH [3]. Coherently, combining Ser and Glycine (Gly) starvation with PHGDH inhibition is much more effective in impairing tumor progression than the sole Ser or Gly limitations in in vitro and in vivo models [7].

In keeping, more recent studies have guessed different non-metabolic roles of PHGDH in cancer. In this line, it has been shown that PHGDH interacts with the translation initiation factors eIF4A1 and eIF4E, supporting the assembly of the complex eIF4F on the 5’ mRNA structure to promote pancreatic cancer progression [8]. Interestingly, PHGDH also promotes the translation of mitochondrial DNA-encoded proteins and sustains respiration in liver cancer cells [9]. Moreover, in glioma cells, PHGDH also interacts and stabilizes FOXM1, thereby inducing the expression of genes involved in cancer progression [10]. In liver cancer, nuclear PHGDH forms a complex with c-Myc, which drives chemokine CXCL1 and IL8 expression, required to recruit neutrophils and tumor associated macrophages to the liver, sustaining tumor progression [11]. In addition, Ma et al., demonstrated that glucose restriction prompts the phosphorylation of PHGDH by p38 at Ser371, sustaining its nuclear translocation. Here, the restrictive effect of PHGDH on NAD^+^ levels represses the PARP1-dependent poly(ADP-ribosyl)ation of c-Jun, thereby suppressing c-Jun transcriptional activity linked to cell growth [12]. In gastric cancer, the interaction of PHGDH with insulin-like growth factor 2 mRNA binding protein 1 (IGF2BP1) stabilizes TCF7L2 mRNA and increases its expression, resulting in the activation of the Wnt/β-catenin signaling pathway thereby contributing to multi-drug resistance [13].

As a part of the combination of therapies (including surgery, chemotherapy, and radiation therapy), 5-Fluorouracil (5-FU) is a primary therapeutic approach for the management of non-resectable colorectal cancer (CRC) and advanced metastatic forms [14]. Despite significant improvements in surgical approaches and adjuvant treatments, the survival rate for CRC patients is still relatively low due to the development of drug resistance. Various underlying mechanisms participate to 5-FU resistance, either intrinsic or acquired, including altered cellular bioavailability and drug metabolism. These mechanisms are mostly driven by various aberrantly expressed genes and proteins [15]. Importantly, the modulation of metabolism-related genes is emerging as a key adaptation, supporting antitumor drug resistance [16]. Specifically, we previously demonstrated that high PHGDH expression correlates with low sensitivity to 5-FU in CRC cells lines [17]. In the present study, we underscored a heterogeneous expression of PHGDH among CRC patients and we confirmed high PHGDH levels in organoids and tumor tissue specimens from patients less responsive to 5-FU-based therapy. Interestingly, performing RNA-seq analysis on PHGDH wild-type and PHGDH-silenced CRC cells, we identified that high PHGDH levels promote the activation of the Hedgehog (HH) signaling, a central pathway during embryonic development, whose deregulation is involved in crucial stages of carcinogenesis in different types of tumors [18]. We demonstrated that HH signaling is a key player in modulating PHGDH-mediated 5-FU response in CRC. Coherently, combining 5-FU with specific HH-signaling inhibitors synergistically limits CRC tumor growth in mice, providing new scenarios for anti-tumor therapeutics designs, to improve the CRC treatment effect and overall patient survival.

## Methods

### Cell culture and treatment

Human colorectal carcinoma cells HCT8, RKO, LS174T, HCT116 were obtained from ATCC and authenticated by the supplier using short tandem repeat (STR) profiling. Tumor cells were maintained in high glucose Dulbecco’s modified Eagles’s medium (DMEM) (Euroclone) supplemented with 10% fetal bovine serum (Euroclone), 1% penicillin and streptomycin (Euroclone) and 1% L-glutamine (Euroclone) in cell culture flasks until 70-80% cell confluence. Cells were grown in a humidified atmosphere with 5% CO_2_ at 37 °C. All cell lines were tested for Mycoplasma by Mycoalert detection kit (Lonza).

HCT116 resistant to 5-FU (HCT116R) were selected by treating HCT116 parental cells with increasing concentrations of 5-FU for six months until the final concentration of 20 μM, as previously reported [17, 19]. HCT116R cells were steadily grown in the presence of 20 μM 5-FU to ensure the maintenance of 5-FU resistance.

5-FU (#F6627) and GANT61 (#G9048) were purchased from Merck Sigma. JC19 was synthetized as previously described [20].

### Cell transfection

HCT8 and LS174T cells were silenced with PHGDH human shRNA plasmid (Origene) or scrambled shRNA (Origene) using Lipofectamine 3000 (Thermo Fisher Scientific) according to the manufacturer’s protocol. After 24 h, transfected cells were treated with 2 μM puromycin for one month to select for stable shPHGDH-expressing clones.

RKO PHGDH overexpressing cells were obtained transfecting cells with plasmid pLHCX expressing the PHGDH cDNA kindly gifted by Sarah-Maria Fendt, VIB-KU Leuven, Belgium. After 24 h transfected cells were treated with 400 μg/mL hygromycin for 1 month until selection of stable PHGDH overexpressing cells.

### Cell transduction

Lentiviral particles for GLI1 silencing were produced in HEK-293T cells as previously described [20]. The shRNA vectors used were pLKO.1-puro (Lv-C) and pLKO.1-puro-shGLI1 (Lv-shGLI1). HCT8 cells were seeded in 6-well plates and transduced with Lv-C or Lv-shGLI1 lentiviruses diluted 1:4 in culture medium in presence of 8 μg/mL polybrene (Merck Sigma). After 24 h the culture medium containing viral particles was replaced with complete medium and after 72 h from the transduction 3,5 μM puromycin was added to select transduced cells. After 5 days of selection, GLI1 protein levels were assessed.

### Western blot analysis

Cells were lysed in RIPA buffer (Thermo Fisher Scientific) and added with Protease Inhibitor and Phosphatase inhibitor (Merck Sigma). Protein lysates were centrifuged at 14,000 rpm, 4 °C for 10 min, and protein concentration were quantified using Bicinchoninic Acid (BCA) assay (Euroclone). Then, 20 μg of total proteins was loaded on SDS-PAGE gels (BioRad) and transferred to PVDF membranes (BioRad). Membranes were blocked for 1 h at room temperature in 5% non-fat dry milk (Santa Cruz Biotechnology) in PBS-Tween 0,1% and then incubated overnight with primary antibody against PHGDH (Thermo Fisher Scientific, #PA5-54,360), GLI1 (Cell Signaling Technology, #2643), c-Myc (Cell Signaling Technology, #5605), phospho-ERK1/2 (Cell Signaling Technology, #9101), ERK1/2 (Cell Signaling Technology, #4348), phospho-AKT (Cell Signaling Technology, #9271), AKT (Cell Signaling Technology, #9272), phospho-P38 (Cell Signaling Technology #4511), P38 (Abcam, ab59461), phospho-H2AX (Cell Signaling Technology, #2577), H3 (Cell Signaling Technology, #9715), H2AX (Cell Signaling Technology, #2595), HSP90 (Santa Cruz Biotechnology, sc-69703), Vinculin (Merck #V9131), β-Actin (Santa Cruz Biotechnology, sc-47778).

Following 1 h incubation at room temperature with anti-rabbit horseradish peroxidase-conjugated (Santa Cruz Biotechnology #2357) or anti-mouse horseradish peroxidase-conjugated (Santa Cruz Biotechnology #516102), membranes were visualized using Clarity Western ECL Substrate (BioRad) and images acquired using Amersham Imager 600 (Amersham). Vinculin, HSP90 or β-Actin was used to ensure equal loading. All western blot images are representative of at least three independent experiments.

### Viability assay

3 × 10^4^ cells/well were seeded in 24-well plates and allowed to adhere for 24 h. Triplicate wells were seeded for each experimental condition. Culture medium was substituted by experimental media. At each endpoint, cells were detached with trypsin (Euroclone), resuspended in DMEM and counted.

### Colony formation assay

After 48 h of 5 μM 5-FU treatment 1 × 10^3^ cells were seeded into six-well plates and cultured for 10 days in complete medium. Then, cells were fixed and stained with Crystal Violet solution. Colonies were photographed. The colonies’ total area was calculated using the ColonyArea plugin of ImageJ imaging system as follow:

$$\mathrm{Colony}\;\mathrm{area}\;\%\;=\;\frac{\mathit\#\mathit\;of\mathit\;pixels\mathit\;in\mathit\;the\mathit\;region\mathit\;with\mathit\;an\mathit\;intensity\mathit\;above\mathit\;zero}{Total\mathit\;\mathit\#\mathit\;of\mathit\;pixels\mathit\;in\mathit\;the\mathit\;same\mathit\;region}\;\times\;100$$

### Invasion assay

Invasion was measured in a Boyden chamber equipped with 8 μm pore filters (Greiner Bio-One) coated with 50 μg/cm^2^ Matrigel (Corning). 8 × 10^4^ cells were detached and resuspended in starvation medium in the upper chamber. In the lower chamber, complete medium containing 10% FBS was added as chemoattractant.

After 16 h, invaded cells were fixed and stained with Crystal Violet. The values for invasion were expressed as the average number of invading cells per microscopic field over five fields.

### Colonsphere formation

Cells were grown in anchoring-independent conditions into poly 2-hydroxyethyl methacrylate (poly-HEMA)-coated dishes (Merck Sigma) with selective serum-free DMEM/F12 medium supplemented with 50X B27 (Gibco), 20 ng/mL bFGF (Bio-Techne), 20 ng/mL EGF (Relia Tech). Briefly, CRC cells were treated with 5-FU for 48 h, then 700 cells/well were seeded in a 96-well plate precoated with poly-HEMA in anchoring-independent conditions. After 7/10 days, photos were taken to determine the number of spheres. Data represents the average number of formed spheres/field, in at least 5 randomly chosen fields. Total colonspheres volume was calculated after measuring with ImageJ length (L) and height (H) using the following formula: V = (L^2^*H)/2, as previously reported [21]. Data represent the average of sphere volume/field, in at least 5 fields. For the evaluation of self-renewal (P2 generation), a single colonsphere was dissociated with Trypsin EDTA and a dilution of one cell per well into 96-well poly-HEMA pre-coated plates was seeded. After 7/10 days, photos were taken to determine the number of spheres performed. The percentage of spheroids was calculated as the number of spheres formed/number of seeded wells.

### RNA isolation and Quantitative Real-Time Polymerase Chain Reaction (qPCR)

Total RNA was extracted from cells or tissue using the RNeasy Plus Mini Kit (Qiagen #74134) according to the manufacturer’s instructions. The amount and purity of RNA were determined with a NanoDrop Microvolume Spectrophotometer and Fluorometer (Thermo Fisher Scientific). cDNA synthesis was obtained by incubating 1 μg of total RNA with High-Capacity cDNA Reverse Transcription Kit (Applied Biosystems) according to the manufacturer’s instructions. qPCR was performed using Luna Universal qPCR Master Mix (New England Biolabs). The qPCR analysis was performed in triplicate with CFX96 Real-Time PCR System (BioRad). Data were reported as relative quantity with respect to the reference sample using the 2^−ΔΔCt^ method. β2-microglobulin was used as reference genes. The specific primers were provided by Thermo Fisher Scientific, see Suppl Table 1.

### Cytosolic and nuclear fraction isolation

Cells were seeded in P100 plates and let to adhere. When dishes became sub-confluent, cells were detached with trypsin and centrifuged at 1000 g for 5 min. Cell pellets were resuspended in a hypotonic solution (10 mM Hepes pH 8, 10 mM KCl, 1,5 mM MgCl, 1 mM DTT, 0,1 mM EDTA) containing 0,2% NP-40, phosphatase and protease inhibitor. The resuspended cell pellet was homogenized by using a glass pestle for 15 min on ice. The solution was centrifuged to pellet nuclei at 16000 g for 10 min at 4 °C. The supernatant (cytosolic fraction) was collected, pellet was lysed with RIPA buffer. Protein content of both fractions was calculated with BCA method. Cytosolic and nuclei purity was confirmed by western blotting analysis with antibodies anti β-Actin or histone H3, respectively.

### Immunoprecipitation

Colon cancer cells (2 × 10^6^) were lysed with RIPA buffer, and 500 μg of nuclear fraction proteins were incubated with specific antibodies at 4 °C overnight, with gentle agitation. Then, A/G protein (Thermo Fisher Scientific) was added to the antibody-lysate complex and incubated for 1 h at 4 °C, with gentle shaking. The complexes were washed with PBS to remove unbound impurities and centrifuged at 14,000 rpm, 10 min at 4 °C. Immunoprecipitated proteins were then resuspended in Laemmli buffer without a reduction agent and separated with SDS page.

### Flow cytometric analysis

Colonspheres, enzymatically dissociated into a single-cell suspension, were resuspended in 100μL PBS and stained with fluorochrome-conjugated antibodies anti-CD133 1:100 (BD Pharmigen, #566596), anti-CD24 1:10 (BD Pharmigen, #560991), anti-CD44 1:50 (BD Pharmigen, #560977) and anti-EpCAM 1:100 (Invitrogen, #17579182) for 30 min at 4 °C in the dark. The cells were then washed with PBS and analyzed using a BD-FACS Canto II flow cytometer. Gating strategies included initial selection of viable cells based on forward scatter (FSC) and side scatter (SSC) parameters, followed by singlet discrimination (FSC-H versus FSC-A). Unstained controls were used to set the background auto-fluorescence and establish gating thresholds for each marker. The mean fluorescence intensity (MFI) was recorded, quantified, and normalized to the experimental control (shCTRL or CTRL). Acquired data were elaborated using BD FACSDiva Software (BD Bioscences).

### Human tissues and organoid generation

Human tissues were obtained from patients undergoing surgery at the Azienda Ospedaliera Universitaria Careggi, Florence, Italy. Written informed consent was obtained from all patients. The study was performed in accordance with the Helsinki Declaration and approved by the ethics committee (RC_AUT_DG_24030_BIO). Tissues were transferred to the laboratory on ice and further processed for molecular analysis and for the generation of organoids as previously described [22–24].

Briefly, for patient-derived cancer organoid (PDCO) generation, biopsies were washed in PBS 1 × with Penicillin/Streptomycin 1% (Euroclone) and 100 μg/ml Primocin (InvivoGen). The tissue was cut into small pieces of 1–2 mm^3^ and digested in 5 mL Advanced DMEM F/12 (Gibco) containing 5 mg/mL Collagenase type IV (Merck Sigma), 10 μL Deoxyribonuclease I from bovine pancreas (2000 Kunitz units/mg, Sigma Aldrich), 2 μm Y27632 dihydrochloride (Tocris) for 1 h at 37 °C pipetting every 10 min. The digested tissue was filtered with a 100 μm cell strainer and DMEM supplemented with 10% fetal bovine serum was added to stop digestion. The digestion mixture was centrifugated for 5 min at 300 RCF. The tube was placed on ice, the pellet was resuspended in Matrigel (Corning) and seeded as drops in 12-well pre-warmed plate. After 20 min, the medium supplemented with growth factor was added. The medium was changed every 2 days. Advanced DMEM F/12 was supplemented with 10 mM HEPES (Euroclone), 2 mM GlutaMax (Gibco), Primocin (InvivoGen), 1 × B-27 supplement (Gibco), 10 mM Nicotidamide (Sigma Aldrich), 1.25 mM N-acetyl-l-cysteine (Cayman), 500 nM A83-01 (Cayman), 50 ng/mL EGF (ReliaTech), 100 ng/mL Recombinant human Noggin (PrepoTech), 500 ng/mL hRSPO1-FC (ImmunoPrecise Antibodies), 10 μM Y27632 dihydrochloride (Tocris), 10 nM Prostaglandin E2 (Selleckchem), 25 ng/mL SB202190 (AdipoGen), 10 mM Gastrin I human (Sigma Aldrich) [25, 26].

### Organoid viability assay

PDCOs were dissociated with Trypsin–EDTA 0,25% (Gibco) and seeded in a 96-well plate at a density of 5 × 10^3^ cells in 7 μL Matrigel droplets. Before treatment, cells were allowed to recover and form organoids for 2 to 3 days. PDCOs were treated with 5-FU, JC19 or in combination and cell viability was assessed after 7 days using CellTiter-Glo 3D reagent (#G9682, Promega). Luminescence was measured. All experiments were performed twice in triplicate.

### Formalin-fixed paraffin embedded (FFPE) organoids

After the generation and growth of the PDCOs, the medium was removed from the well, and 150 μL of 2 mg/mL Dispase II (Merck Sigma) was added to each well. After 1 h of incubation at 37 °C 1 mL of PBS 1X was added. The drops were collected into a 15 mL Falcon tube, filled with PBS 1X and centrifuged at 300 RCF at room temperature for 5 min. The supernatant was discarded, and the pellet was stored at 4 °C in 1 mL of 4% formalin. The fixed PDCOs were resuspended in 80 µL of pre-warmed HistoGel (Epredia) and placed as a single drop on parafilm. After solidification (1 h) the HistoGel drop was transferred to a tissue cassette for paraffin embedding.

### Immunohistochemistry (IHC) analysis

Human- or mouse-derived tumors were fixed in 4% formalin and subsequently embedded in paraffin wax. 7 μm tissue sections of paraffin-embedded of PDCOs, human- or mouse-derived tumors were prepared for histology and IHC analysis. Hematoxylin & Eosin (H&E, #3801698, Leica Biosystems) staining was performed on individual specimens. Human-derived tumor (#CRC) and PDCO sections were immunostained using an antibody specific to PHGDH (1:1000, #PA5-54360 Invitrogen, Waltham, Massachusetts), while mouse-derived tumor sections were stained with Ki67 (#GA626, Dako Omnis) antibody. IHC was performed using the Leica BOND-MAX automated staining system. Staining was developed with 3,3’-diaminobenzidine (DAB) and counterstained with Hematoxylin (#DS9800, Leica Biosystems). Digital images were acquired with an Aperio LV1 slide scanner and analyzed using ImageScope software.

Archive formalin fixed paraffin embedded (FFPE) 3 µm tissue sections from the Section of Pathological Anatomy, University of Florence, were prepared for immunohistochemical analysis. Sample processing was performed with an automated immunostainer (Ventana Discovery ULTRA, Ventana Medical Systems, Tucson, AZ). Sections were deparaffinized in EZ prep (#950–102; Ventana Medical Systems, Tucson, AZ), antigen retrieval was achieved by incubation with cell-conditioning solution 1 (CC1) (#950–124; Ventana Medical Systems, Tucson, AZ). Sections were then incubated with antibody anti-PHGDH (#PA5-54360,1:500 Rabbit polyclonal; Invitrogen, Waltham, Massachusetts). The signal was developed with Ultra Map diaminobenzidine anti-mouse or anti-rabbit HRP detection kit (Ventana Medical Systems, Tucson, AZ). The score of PHGDH expression was classified into 4 categories: 0 no staining, 1 + weak staining, 2 + moderate staining, 3 + strong staining. The product of staining intensity score and the percentage of positive tumor cells (0% to 100%) was considered as the final score of PHGDH expression, which ranged from 0 (no staining) to 300 (100% of cells with 3 + staining intensity) [27]. Quantification was conducted by analyzing three representative fields for each CRC sample, with expression levels reported as Cytoplasm H-score mean values. H-scores were stratified into low and high-expression groups based on the overall mean value across all analyzed CRC samples.

### In vivo experiment

Animal experiments were performed in accordance with national guidelines and approved by the ethical committee of the Animal Welfare Office of Italian Health Ministry. All procedures conformed to the legal mandates and the Italian guidelines for of laboratory animals. All animals received human care, and study protocols comply with the institution's guidelines. Studies involving animal experiments conform to the Animal Research: Reporting of In Vivo Experiments (ARRIVE) guidelines (http://www.nc3rs.org.uk/arriveguidelines), developed by the National Centre for the Replacement, Refinement and Reduction of Animals in Research (NC3Rs) to improve standards and reporting of animal research.

Female Fox1 nu/nu mice (Charles River Laboratories International) were injected subcutaneously into both flanks with 8 × 10^6^ HCT8 cells in 100 μl PBS. When tumors were palpable, mice were randomized and treated for two weeks with vehicle (PBS, 5% ethanol/PBS, 10% DMSO), 5-FU (25 mg/kg in PBS, 5% ethanol) with a cyclic regimen composed of three daily injections followed by two recovery days, JC19 (15 mg/kg in PBS, 10% DMSO) twice a day or with a combination of the two treatments. All treatments were carried out by intraperitoneal injection (IP). Treatment doses were adjusted to mouse weight with an injection volume of a maximum of 100 μL. Animals (6 per group) were monitored daily. Tumors were measured by caliper, and average tumor volume was calculated using the formula: volume = length x width^2^/2. Humane endpoints were determined according to the parameters indicated in the project authorized by the Italian ethical committee of Animal Welfare Office of Italian Work Ministry (authorization number: n° 981/2024-PR), in particular the maximal tumor volume permitted by our ethics committee was 1200 mm^3^. The tumor volume does not exceed this limit at any point during the study.

### Ultrasound and photoacustic imaging

At the endpoint of the in vivo experiment tumor volumes were determined with high-frequency ultrasound (HF-US) imaging using Vevo F2 LAZR-X system (Fujifilm Visualsonics) performing 3D acquisition in B-Mode on live mice. During the acquisitions, mice were anesthetized with a continuous flow of isoflurane (initial induction at 4% and maintenance at 2%) and placed on a mouse handling table, heated at 37 °C, in prone position. Respiration rate and body temperature ECG were monitored during the analysis. 55-MHz transducer was used for echography. Data obtained were analyzed using Vevo LAB software (Fujifilm Visualsonics) to measure the tumor volumes.

### Library preparation and RNA-sequencing

Total RNA was isolated from cells using the RNeasy Plus Mini Kit (QIAGEN) according to the manufacturer's protocol. Universal Plus mRNA-Seq kit (Tecan Genomics, Redwood City, CA) has been used for library preparation following the manufacturer’s instructions (library type: fr-secondstrand). RNA samples were quantified and quality tested by Agilent 2100 Bioanalyzer RNA assay (Agilent technologies, Santa Clara, CA). Final libraries were checked with both Qubit 2.0 Fluorometer (Invitrogen, Carlsbad, CA) and Agilent Bioanalyzer DNA assay. Libraries were then prepared for sequencing and sequenced on paired-end 150 bp mode on NovaSeq 6000 (Illumina, San Diego, CA).

### Computational analyses

FastQC was employed to perform a comprehensive quality assessment on all fastq files ensuring that the sequencing data met predefined quality standards prior to downstream analyses. Transcript abundance was estimated using Salmon [28], a widely used tool for accurate and bias-aware RNA-seq quantification. The latest version of the human reference transcriptome (hg38) was used for this purpose. Following transcript quantification, raw counts were imported into the R environment using the tximport package [29]. Differential expression analysis was then performed using the DESeq2 package [30] in R. The results from Wald test were utilized to rank the differentially expressed genes (DEGs). To further explore biological themes associated with the identified DEGs, gene set enrichment analysis (GSEA) was conducted using the most recent version of the fGSEA R’packages and the most updated HALLMARK msig gene sets [31]. Following the recommendations of the GSEA software authors, all the results with a False Discovery Rate (FDR) < 0.25 were considered statistically significant.

RNA-seq and clinical data from colorectal adenocarcinoma patients were obtained from The Cancer Genome Atlas (TCGA) database, specifically from the TCGA-COAD (colon adenocarcinoma) and TCGA-READ (rectal adenocarcinoma) projects. Only patients with documented 5-FU-based chemotherapy treatment were included in the analysis. Patients were stratified into responders (R) and non-responders (NR) based on clinical outcomes following treatment. The expression data in the TCGA database had been previously calculated using STAR alignment and quantified with featureCounts. The expression profiles were imported into R, and raw counts were processed following standard DESeq2 procedures for normalization and differential expression analysis. Gene Set Enrichment Analysis was subsequently performed using the fGSEA package with the HALLMARK gene sets from MSigDB to identify significantly enriched pathways between the R and NR groups.

The analysis of the correlation between PHGDH mRNA expression and overall survival (OS)/Relapse Free Survival (RFS) in CRC patients was performed using the open-access database Kaplan–Meier plotter (http://www.kmplot.com). Expression of PHGDH in normal, primary tumors and metastases was obtained from the The Cancer Genome Atlas data set on TNM plot database (http://www.tnmplot.com). The classification into low and high PHGDH was performed by the"Auto select best cutoff"option, where the lower and upper quartiles define the range for all possible cutoff values. The threshold that yields the best performance within this range is selected as the cutoff.

### Statistical analysis

Statistical analysis of the data was performed by unpaired Student t-test or One-way ANOVA for pairwise comparison of groups with GraphPad Prism version 8.0 (GraphPad Software). All data were expressed as the mean ± SEM. A *p* value ≤ 0.05 was considered statistically significant. All the statistical analyses were carried out on three biological replicates. Levene’s test for equality of variance was performed to assess the heterogeneity in PHGDH expression in tumor and healthy patients’ cohort. The significance level was considered at 5%. Bliss independence analysis was used to evaluate the effects of drug combinations. Bliss score: = 0, no functional interaction; > 0, synergism; < 0, antagonism.

## Results

### PHGDH expression is heterogeneous in CRC tissues and in patient-derived 3D organoids

**Fig. 1:**
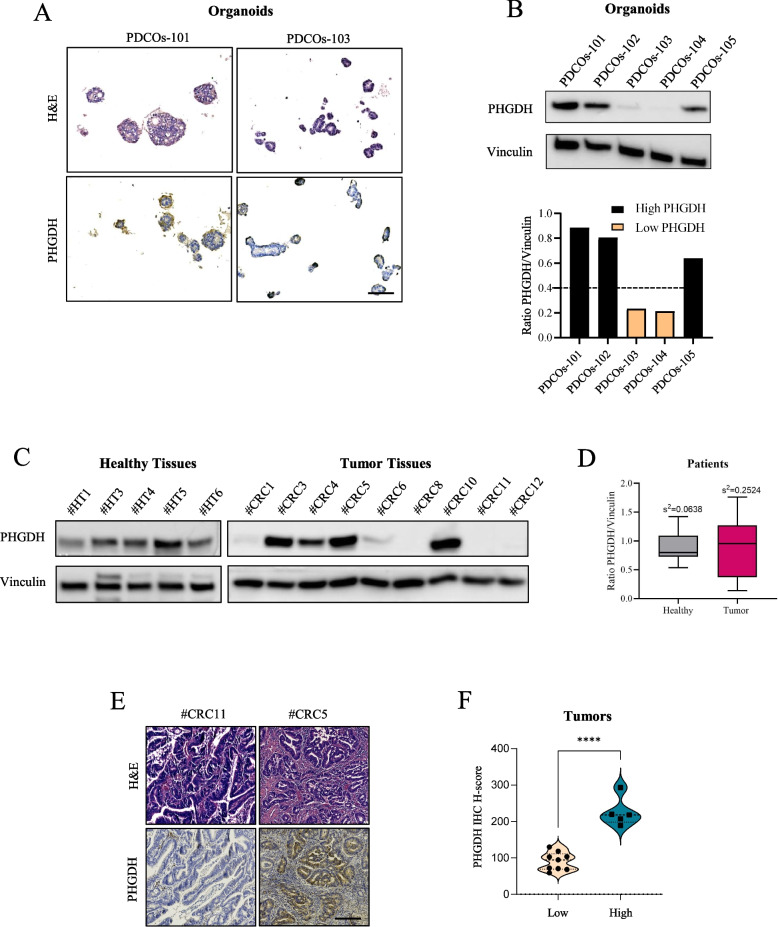
PHGDH expression is heterogeneous in CRC tissues and in PDCOs.** A** Representative H&E and PHGDH staining of PDCOs by IHC detection (left, high PHGDH; right, low PHGDH) (magnification 20 ×, scale bar: 100 μm). **B** PHGDH protein expression levels in PDCOs (western blot and relative bargraph, showing quantification of PHGDH protein levels). Vinculin was used as loading control. The dashed line represents an arbitrary threshold used to cluster the organoids into high and low PHGDH. Western blot image is representative of at least three independent experiments. **C** PHGDH protein expression levels in healthy and tumor tissues from CRC patients’ specimens. Western blot of representative samples within the cohort. Vinculin was used as loading control. **D** Box and Whisker plot comparing the expression distribution of PHGDH in healthy (*n* = 19) and tumor (*n* = 24) CRC tissues. Values of variance of the two datasets are reported at the top of each box (healthy, s^2^ = 0.0638; tumor, s^2^ = 0.2524). Levene’s test for equality of variance resulted in a f-ratio value of 14.86044 and a *p*-value of 0.0004, indicating that the variances of the two populations are significantly different from each other. **E** Representative images of H&E and low (left) and high (right) PHGDH IHC staining on human-derived tumors (magnification 10 ×, scale bar: 200 μm), along with the corresponding quantification (PHGDH H-score) across 14 different tumor samples (**F**). Tumors are categorized into low and high PHGDH expression groups based on H-score values. t-test **** *p* < 0.0001

Notably, PHGDH protein level is a dynamic feature that can be modulated over time during cancer progression [32]. Previous findings from our laboratory pointed out a high variability in PHGDH expression among different CRC cell lines, demonstrating that higher PHGDH levels correlate with lower sensitivity to 5-FU treatment [17]. This aspect led us to hypothesize that differential basal protein levels and dynamic regulation of PHGDH during the treatment with 5-FU could drive the evolution of drug resistance in CRC patients. First, we investigated this hypothesis in patient-derived cancer organoids (PDCOs) from freshly isolated CRC samples. Interestingly, PDCOs from different patients displayed distinct PHGDH levels proving the existence of a great variability within different samples as shown by representative IHC images in Fig. 1A and western blot analysis (Fig. 1B upper). This evidence allowed us to classify PDCOs as “high PHGDH” and “low PHGDH” (Fig. 1B down).

Accordingly, by investigating PHGDH levels in tumor tissues and healthy counterparts of surgical explants from different CRC patients (Suppl. Table 2), we confirmed that PHGDH expression is highly heterogeneous in tumor specimens while it is more homogeneous across healthy samples (Fig. 1C-D). By performing H&E and immunohistochemistry (IHC) staining of PHGDH on tumor tissue samples from the same CRC patients we confirmed the heterogeneity in PHGDH levels across patients (Fig. 1E and Suppl. Table 2). The quantification of PHGDH, as measured by H-score, revealed significant variability between different tumors, allowing to categorize patients into “low” and “high PHGDH” expression groups (Fig. 1F). This aspect led us to hypothesize that the diversity among CRC patients in basal PHGDH protein levels and its dynamic regulation along the treatment with 5-FU could result in differential therapy efficacy, finally influencing cancer progression and patients’ outcome. Intriguingly, although most of the follow-ups for this recently collected cohort of patients are still unavailable, we noticed that all the four patients who have developed recurrences to date (see Suppl. Table 2) exhibit higher PHGDH levels with respect to representative non-relapsing patients (Suppl. Fig 1). This evidence suggested that elevated levels of PHGDH could correlate with a worse prognosis and prompted us to examine a broader dataset of CRC patients derived tissue samples.

### PHGDH sustains resistance to 5-FU in CRC patients

**Fig. 2:**
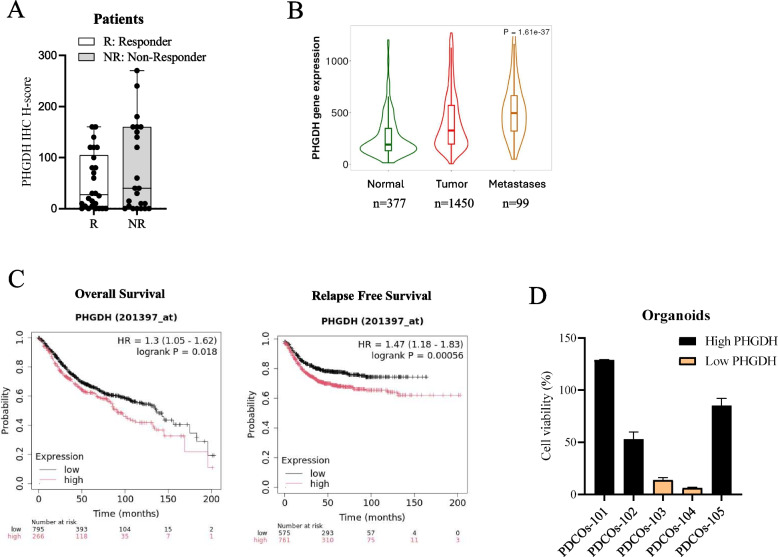
PHGDH expression correlates with resistance to 5-FU in CRC patients and PDCOs.** A** Retrospective analysis of 50 CRC patients showing the correlation between PHGDH expression level and therapy response (R: Responder, NR: Non Responder).** B** Violin plots show PHGDH gene expression profile in normal colon (Normal), primary (Tumor) and metastases (Metastases) of colon cancer obtained by online analysis platform on TNMplot. **C** Kaplan–Meier plotter of high and low PHGDH expression in CRC. OS and RFS in CRC patients expressing high PHGDH (red) and low PHGDH (black) levels. **D** PDCOs response to 5 μM 5-FU treatment. Cell viability was assayed after 7 days of treatment by CellTiter-Glo 3D reagent

Thus, to verify the role of PHGDH expression in patients’ clinical outcomes, we performed a retrospective analysis on 50 biopsies collected during the previous 10 years from patients with advanced CRC who received post-surgery 5-FU-based therapy (see Suppl. Table 3). Thus, we investigated a possible correlation between PHGDH expression and chemotherapy response. To this aim, we classified patients as responders (R) and non-responders (NR) based on clinical outcomes of at least the last 5-years. The final score of PHGDH immunostaining was obtained by the product of staining intensity score (0, 1 +, 2 +, and 3 +) and the percentage of positive tumor cells (0% to 100%). Interestingly, a clear enrichment of high-PHGDH samples in the NR group emerged with respect to the R one, indicating a positive correlation between PHGDH expression and 5-FU resistance (Fig. 2A).

Moreover, the analysis of public databases (https://www.cbioportal.org/) showed that CRC tumor samples and metastases display higher levels of PHGDH with respect to their normal counterparts, suggesting that increased PHGDH expression is associated with a poor prognosis in CRC patients (Fig. 2B). Indeed, the prognostic value of PHGDH expression in CRC patients was further assessed according to overall survival (OS) and relapse-free survival (RFS) using the Kaplan–Meier plotter. Concordantly with our results, the plot shows that high PHGDH expression correlates with poor OS (*P* < 0.05, HR = 1.3) as well as poor RFS (*P* < 0.001, HR = 1.47) in CRC patients (Fig. 2C).

Finally, the correlation between PHGDH and 5-FU sensitivity was also confirmed in CRC PDCOs. A significant difference in the response to 5-FU was observed across samples (Fig. 2D) and, in agreement with data shown above, the higher sensitivity of PDCOs to 5-FU correlated with a lower expression of PHGDH (Fig. 1B and Fig. 2D).

Collectively, these data demonstrate that PHGDH expression is highly heterogeneous in patients’ CRC samples, and its levels may be predictive of patient’s response to 5-FU based chemotherapy.

### PHGDH promotes CRC cell aggressiveness

#### PHGDH promotes resistance to 5-FU treatment in CRC cell lines

**Fig. 3:**
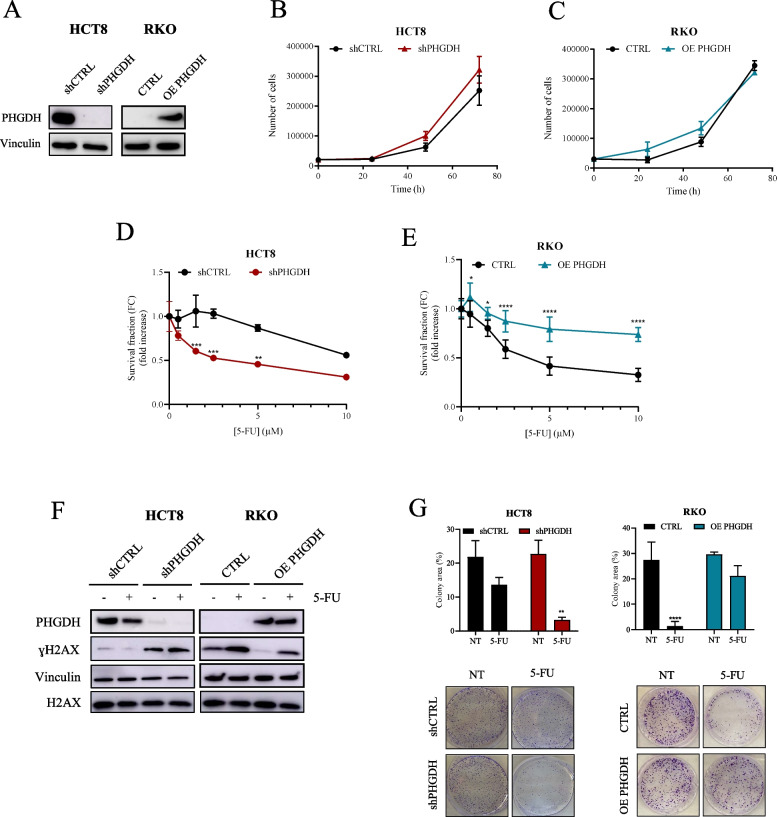
PHGDH supports resistance to 5-FU.** A** PHGDH protein levels in HCT8 and RKO cells following shRNA silencing or overexpression, respectively. Vinculin immunoblot was performed to ensure equal loading. **B-C** Proliferation rate of HCT8 and RKO cells after stable transfection. Data are reported as mean ± SEM from three independent experiments. **D-E** Survival fraction of HCT8, HCT8 shPHGDH, RKO and RKO OE PHGDH treated with increasing concentration of 5-FU for 48 h. Data are reported as mean ± SEM from three independent experiments; t-test; * *p* < 0.05, ** *p* < 0.01, *** *p* < 0.001, **** *p* < 0.0001. **F** γH2AX levels in transfected and parental HCT8 and RKO cells. H2AX and vinculin were used as controls. Each image is representative of at least three independent experiments. **G** Colony formation assay of HCT8, HCT8 shPHGDH, RKO and RKO OE PHGDH cells grown either in the absence or presence of 5 μM 5-FU. After 48 h, cells were detached, counted, and 1000 cells were plated in new dishes, and incubated for at least 10 days. Formed colonies were stained with Crystal Violet. Representative images are shown. Data are reported as mean ± SEM from three independent experiments; t-test; ** *p* < 0.01, **** *p* < 0.0001

To confirm the relevance of PHGDH in conferring 5-FU resistance, we adopted loss of function and gain of function approaches to modulate PHGDH levels on high- and low-PHGDH cell lines respectively. Specifically, PHGDH silencing was used in high-PHGDH cells (HCT8) while PHGDH overexpression was used in low-PHGDH cell lines (RKO) [17] (Fig. 3A).

First, we demonstrated that PHGDH levels do not interfere with cell proliferation under basal conditions as both PHGDH silenced HCT8 cells (HCT8 shPHGDH) and RKO cells overexpressing PHGDH (RKO OE PHGDH) proliferate similarly to their wild-type counterparts (Fig. 3B-C). Conversely, cell viability under 5-FU treatment is strongly affected by the expression of PHGDH (Fig. 3D-E), according to previously published results [17].

In line, the quantification of histone H2AX phosphorylation (γH2AX), a known marker of DNA double-strand break, proved that PHGDH expression confers increased ability to counteract DNA damage accumulation upon 5-FU treatment. Specifically, we revealed lower levels of γH2AX in RKO OE PHGDH compared to wild-type counterpart both in basal conditions and following stimulation with 5-FU, while HCT8 shPHGDH cells display increased accumulation of DNA damage mainly in basal and 5-FU treated conditions (Fig. 3F). Similar results were obtained in LS174T cells silenced for PHGDH (LS174T shPHGDH), showing increased sensibility to 5-FU and up-regulated levels of γH2AX with respect to the parental cells (Suppl Fig. 2A-C). These data strongly sustain the expression of PHGDH is crucial to promote CRC aggressiveness sustaining 5-FU resistance by supporting DNA damage repair. In addition, we demonstrated that high levels of PHGDH confer a more aggressive phenotype to CRC cells as shown by increased colony-forming ability after 5-FU treatment in RKO OE PHGDH cells with respect to RKO, besides HCT8 shPHGDH cells are more sensitive to 5-FU toxic effect than parental cells and hence exhibit a decreased ability to form colonies (Fig. 3G).

#### PHGDH promotes invasion and stemness-properties acquisition in CRC cell lines

**Fig. 4:**
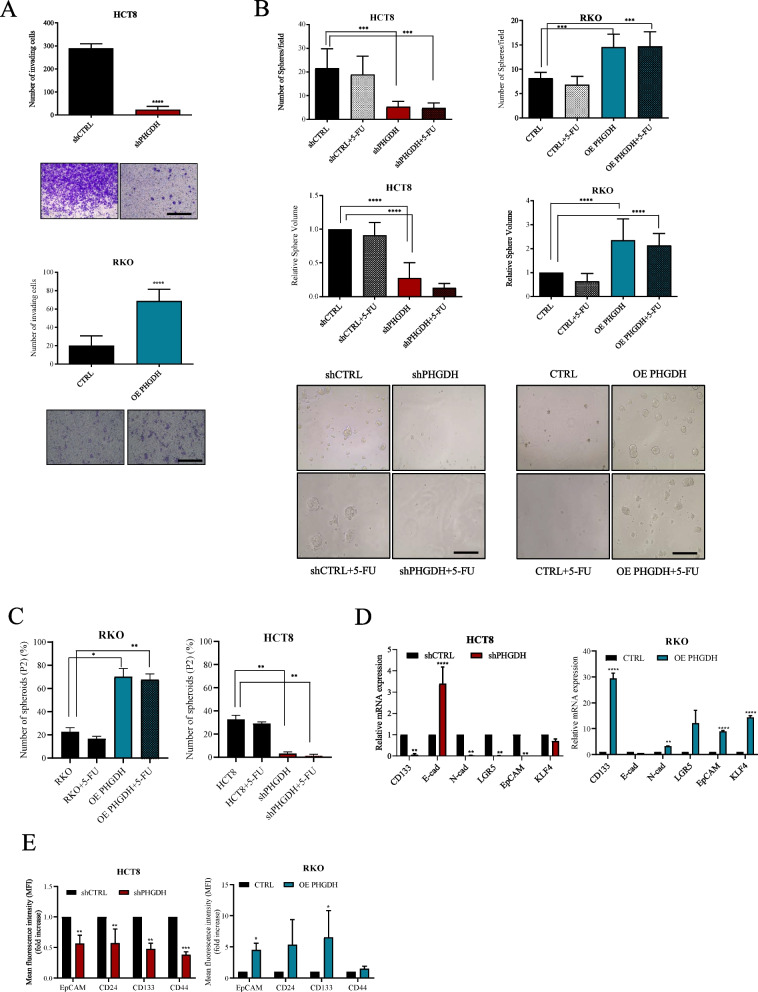
PHGDH confers aggressiveness to CRC cells. **A** Invasive abilities of PHGDH-silenced or PHGDH-overexpressing cells. CRC cells were seeded in the upper compartment of 8 μm Transwell system coated with Matrigel. After 16 h invaded cells were stained with Crystal Violet and counted. Representative images of invaded CRC cells are shown below the bargraphs (magnification 20 ×, scale bar: 100 μm). Data are reported as mean ± SEM from three independent experiments; t-test; **** *p* < 0.0001. **B** PHGDH silenced or overexpressing cells were treated with 5 μM 5-FU for 48 h then P1 spheres forming ability was performed seeding 700 cells/well in 96-well plate. After 7 days colonspheres were counted, and volumes were calculated as described in Methods. Representative images of CRC spheroids are shown below the bargraphs (magnification 20 ×, scale bar: 100 μm). Data are reported as mean ± SEM from three independent experiments; bargraph represents the fold increase relative to respective CTRL; t-test; *** *p* < 0.001, **** *p* < 0.0001. **C** Cells disaggregated from P1 spheres were re-plated to test secondary spheroid (P2) formation ability in HCT8, HCT8 shPHGDH cells, RKO and RKO OE PHGDH. Data are reported as mean ± SEM from three independent experiments; t-test; * *p* < 0.05, ** *p* < 0.01. **D** mRNA expression levels of genes involved in CSC phenotype acquisition and EMT program in P1 colonspheres. Data are reported as mean ± SEM from three independent experiments; t-test; ** *p* < 0.01, *** *p* < 0.001, **** *p* < 0.0001. **E** Membrane EpCAM, CD24, CD133 and CD44 expression levels in CRC cells evaluated in P1 colonspheres by flow cytometry. Data are reported as mean ± SEM from three independent experiments; t-test; * *p* < 0.05, ** *p* < 0.01, *** *p* < 0.001

Together with acquiring a drug-resistant phenotype, the increased invasive ability and stemness properties are known hallmarks of tumor cell progression [33, 34]. In accordance, we demonstrated that modulating PHGDH levels affects CRC cell invasion. Specifically, RKO OE PHGDH cells display increased invasive ability compared with their wild-type counterpart, while silencing of PHGDH induces the opposite effect (Fig. 4A; Suppl Fig. 2D). Furthermore, to investigate whether PHGDH expression modulation affects stemness properties, we performed the following assays: i) assessment of colonspheres formation (P1 generation) and secondary colonspheres formation (P2 generation) to test the self-renewal properties of primary generated P1 spheroids; ii) evaluation of the expression of key stemness genes by qPCR, and iii) quantification of stemness markers expression on the cell surface by flow-cytometry. By generating P1 colonspheres following 5-FU treatment, we observed that both the number and dimension of P1 colonspheres correlate with PHGDH expression (Fig. 4B). Moreover, by investigating cell ability to generate secondary colonspheres by serial passages, we noticed that the presence of high levels of PHGDH positively affects CRC cells self-renewal ability as demonstrated by increased the number of P2 colonspheres in RKO OE PHGDH with respect to control cells even following 5-FU treatment, whereas lack of PHGDH profoundly impairs the self-renewal ability of HCT8 cells both in basal and 5-FU treatment conditions (Fig. 4C). Furthermore, qPCR analysis performed on P1 colonspheres derived from RKO OE PHGDH or HCT8 cells express high level of stemness- and epithelial-mesenchymal transition (EMT)-related genes respect to the relative counterpart expressing low levels of PHGDH (Fig. 4D).

Besides, by evaluating the expression levels of cancer stem cell (CSC) surface markers, EpCAM, CD24, CD133 and CD44 by flow cytometry, we confirmed a direct correlation between stemness markers levels and PHGDH expression in HCT8 and RKO P1 colonspheres (Fig. 4E). Collectively, these results suggest that PHGDH expression is involved in increased cancer aggressiveness and promotes an enrichment in the stemness properties of CRC cell lines.

### PHGDH silencing decreases Hedgehog pathway and resistance to 5-FU

**Fig. 5:**
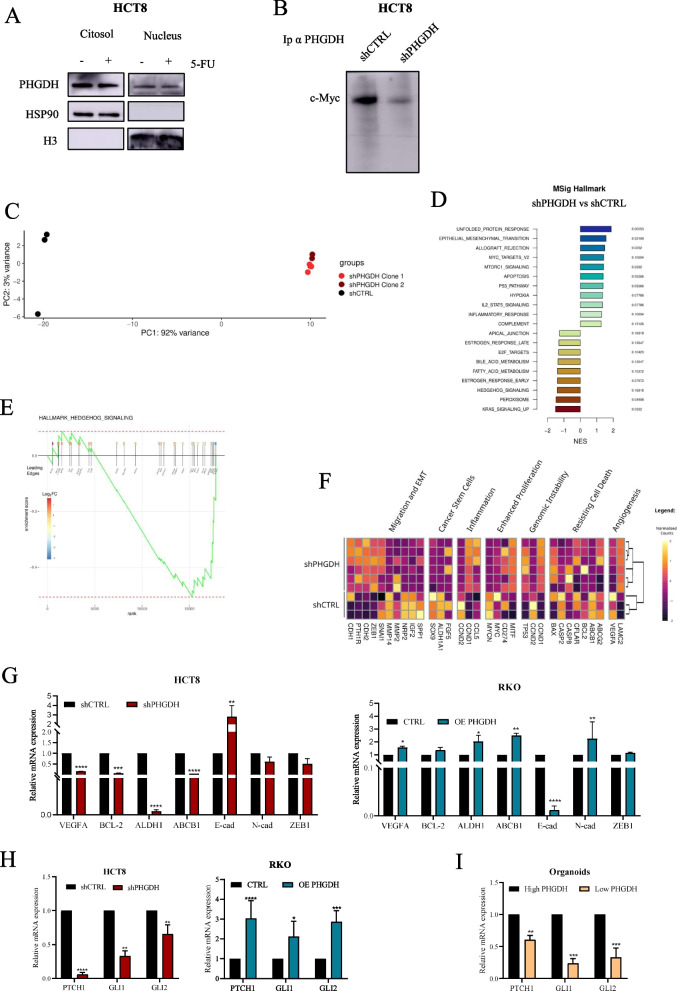
PHGDH modulation affects Hedgehog signaling pathway.** A** PHGDH protein level in cytosolic or nuclear compartment in HCT8 cells treated with 5 μM 5FU for 48 h. HSP90 and H3 were used as loading control for cytosolic and nuclear compartment, respectively. **B** Co-immunoprecipitation of PHGDH and c-Myc in shCTRL or shPHGDH cells. An anti-PHGDH antibody was used to pull down PHGDH, then western blotting for c-Myc was performed. **C** PCA displays the distribution of samples based on their gene expression profiles, projected onto the first two principal components (PC1 and PC2). PC1 (x-axis) captures 92% of the total variance, while PC2 (y-axis) captures 3%. Samples are color-coded according to their group: black points indicate shCTRL replicates, red and brown points indicate shPHGDH replicates. **D** Pathway Enrichment Analysis of Differentially Expressed Genes illustrating the top enriched pathways in the comparison between shCTRL and shPHGDH samples. The analysis was performed using the MSigDB Hallmark gene set. The x-axis represents the *Normalized Enrichment Score (NES)*, which indicates the up/down-regulation degree of the altered pathways: Pathways with positive *NES* values are up-regulated in shPHGDH samples compared to shCTRL, while pathways with negative NES values down-regulated. The associated adjusted *p*-values (Benjamini-Hochberg) are displayed to the right of each bar. **E** GSEA focused on the Hallmark HH Signaling pathway. The plot displays the resulting normalized enrichment score (NES, green line) of the ranked list of DEGs across the dataset. The leading edge, i.e. the genes contributing most significantly to the enrichment, are displayed below each bar which represents the position of the gene in the ranked list. Each gene is also annotated by the log2FC (boxes above the bars), providing an indication of its expression change in the comparison. The red dashed lines indicate the maximum and minimum NES achieved. **F** Heatmap of HH Pathway Target Genes: displays the expression levels of HH pathway target genes across different samples. The genes are organized based on their involvement in specific cellular processes annotated on the top. Sample clustering is shown via a dendrogram at the top of the heatmap. **G** mRNA expression levels of genes involved in HH pathway analyzed by qPCR. Data are reported as mean ± SEM from three independent experiments; t-test; * *p* < 0.05, ** *p* < 0.01, *** *p* < 0.001, **** *p* < 0.0001. **H-I** mRNA expression levels of PTCH1, GLI1 and GLI2 in PHGDH-silenced cells or PHGDH-overexpressing and PDCOs analyzed by qPCR using parental cells or high PHGDH organoids as comparator. Data are reported as mean ± SEM from three independent experiments; t-test; * *p* < 0.05, ** *p* < 0.01,*** *p* < 0.001, **** *p* < 0.0001

Recently, a non-metabolic function of PHGDH has been highlighted in cancers, often dependent on its nuclear localization [11, 12, 35, 36]. In this context, we highlighted that PHGDH, as already reported by others, exhibits a nuclear localization in HCT8 cells (Fig. 5A). Given the increasing evidence that nuclear PHGDH can modulate transcriptional programs by interacting with key transcription factors [10–12], we explored its potential association with c-Myc, a master regulator of cell proliferation and metabolism, frequently dysregulated in CRC [37, 38]. Moreover, coimmunoprecipitation experiments suggest that PHGDH and c-Myc associate at nuclear level in HCT8 cells (Fig. 5B), as previously reported by Zhu H et al., [11], and support the hypothesis that PHGDH may exert its catalytically independent functions through interaction with the transcriptional machinery.

Thus, to elucidate a possible non-metabolic functional role of PHGDH in therapy resistance in CRC, we performed RNA-seq analysis on HCT8 cells and two independent PHGDH-silenced HCT8 (HCT8 shPHGDH) clones. Principal Component Analysis (PCA) demonstrated robust reproducibility among biological replicates and distinct clustering between experimental conditions (Fig. 5C).

Pathway Enrichment Analysis revealed significant upregulation of pathways associated with unfolded protein response, mTORC1 signaling, hypoxia response, and p53 signaling in HCT8 shPHGDH cells compared to parental cells. Conversely, among the most down-regulated pathways in silenced cells compared to control cells, we observed the Hedgehog (HH) signaling (Fig. 5D). HH signaling has a key role in maintaining stemness and promoting chemoresistance across multiple cancer types [39–41]; in particular, given the established role in conferring resistance to 5-FU treatment, we decided to focus our subsequent analyses on clarifying the relationship between HH, PHGDH and therapy resistance.

We next performed a focused pathway analysis to characterize the alteration of the HH signaling upon PHGDH modulation. Consistent with results obtained by Gene Set Enrichment Analysis (GSEA) (Fig. 5E), the expression of multiple HH pathway target genes decreased in PHGDH-silenced cells (Fig. 5F). To further validate these results, we confirmed the modulation in the expression of relevant HH target genes in HCT8 shPHGDH and RKO OE PHGDH cells by qPCR. Coherently, we identified a downregulation in the expression of HH targets in HCT8 shPHGDH cells compared to wild-type ones and an up-regulation in RKO OE PHGDH with respect to RKO cells (Fig. 5G), endorsing the correlation between PHGDH expression and HH signaling.

Moreover, we corroborated the link between HH and PHGDH by confirming a decreased expression in the final effectors of the HH pathway, Glioma-associated oncogene 1 (GLI1), Glioma-associated oncogene 2 (GLI2) and the specific key player of HH pathway Patched1 (PTCH1) in HCT8 shPHGDH cells compared to controls. Conversely, increased expression of GLI1, GLI2 and PTCH1 was confirmed in RKO OE PHGDH cells with respect to parental ones (Fig. 5H). To further establish the relationship between PHGDH and HH signaling, we then compared the expression of these transcripts in high-PHGDH (LS174T) and low-PHGDH (RKO) cells. qPCR analysis confirmed previously obtained results (Suppl. Figure 3A-B). Coherently, in patients’ derived 3D organoids, we detected a close correlation between the expression of PHGDH and genes related to HH pathway (Fig. 5I). Collectively, these analyses consistently demonstrate that PHGDH positively modulates HH signaling pathway activation.

### HH pathway inhibition increases sensitivity to 5-FU in CRC cells and patient-derived 3D organoids

Interestingly, previous studies underscored that HH pathway contributes to stemness and drug resistance in different tumors [42, 43]. Notably, the activation of HH signaling is a key-driver of resistance to 5-FU in LoVo cells and GLI1 and GLI2 knockdown sensitizes gastric cancer and CRC cells to 5-FU [44, 45]*.*

**Fig. 6:**
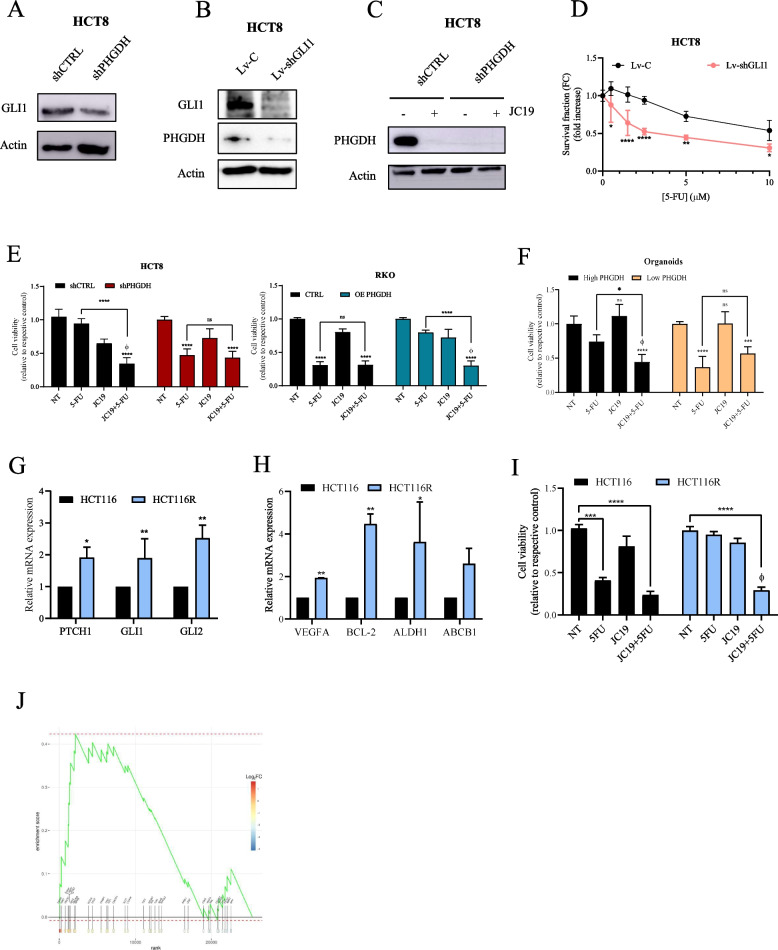
GLI1 inhibition resensitizes CRC cells to 5-FU treatment. **A** GLI1 protein level in shCTRL or shPHGDH cells. β-Actin immunoblot was performed to ensure equal loading. **B** Representative images of western blot for GLI1, PHGDH and β-Actin of HCT8 cells silenced with GLI1 shRNA.** C** Immunoblot of PHGDH in HCT8 shCTRL or shPHGDH cells treated with 2 μM JC19 for 48 h. β-Actin immunoblot was performed to ensure equal loading. **D** Effects of 5-FU on GLI1-silenced HCT8 cells. Cells were treated for 48 h with increasing concentration of 5-FU. Data are reported as mean ± SEM from three independent experiments; t-test; * *p* < 0.05, ** *p* < 0.01, **** *p* < 0.0001. **E–F** Survival fraction of transfected cells (E) and PDCOs (F) treated or not with 5 μM 5-FU in combination with 2 μM JC19 for 48 h (CRC cells) or 7 days (PDCOs). Data are reported as mean ± SEM from three independent experiments; ANOVA; * *p* < 0.05, *** *p* < 0.001, **** *p* < 0.0001. ϕ indicates synergistic effect (Bliss test > 0) with respect to single treatments. **G** PTCH1, GLI1 and GLI2 mRNA expression level in HCT116 and HCT116R cells. HCT116 was used as comparator. Data are reported as mean ± SEM from three independent experiments; t-test; * *p* < 0.05, ** *p* < 0.01. **H** mRNA expression levels of HH pathway target genes analyzed by qPCR. HCT116 was used as comparator. Data are reported as mean ± SEM from three independent experiments; t-test; * *p* < 0.05, ** *p* < 0.01. **I** Survival fraction of HCT116 and HCT116R cells treated or not with 5 μM 5-FU in combination with 2,5 μM JC19 for 48 h. Data are reported as mean ± SEM from three independent experiments; ANOVA; *** p < 0.001, **** *p* < 0.0001. ϕ indicates synergistic effect (Bliss test > 0) with respect to single treatments. **J** GSEA of HH in R *vs* NR colon cancer patients from publicly available TCGA datasets (TCGA-READ, TCGA-COAD)

Indeed, we demonstrated that PHGDH silencing in HCT8 cells reduces GLI1 mRNA and protein levels (Fig. 5H and 6A). Interestingly, GLI1 silencing (Fig. 6B) and its pharmacological inhibition with JC19, a 4-methoxy-8-hydroxyquinoline able to impair GLI1/2 transcriptional activity interfering with their binding to DNA [20] (Fig. 6C) reduce PHGDH expression level in HCT8 cells, resulting in increased sensitivity to 5-FU administration (Fig. 6D). These data highlighted a positive feedback loop between GLI1 and PHGDH, suggesting that pharmacological inhibition of GLI factors, through the administration of JC19 or GANT61 [20, 46], could improve the sensitivity of CRC cell lines to 5-FU administration.

Really, we demonstrated that combining 5-FU and JC19 (Fig. 6E) or GANT61 (Suppl. Figure  3 C) is effective in enhancing the chemo-sensitivity of CRC cells and patients’ derived 3D organoids (Fig. 6F). Of note, the effect of the combo treatment is synergic (Bliss > 0) in cell lines and organoids expressing high PHGDH levels (HCT8 and RKO OE PHGDH) (Fig. 6E-F), whereas in low PHGDH expressing cells and organoids JC19 does not potentiate the effect of 5-FU (Fig. 6E-F).

All together these data suggest that HH pathway inhibitors increases 5-FU chemosensitivity in CRC cells and organoids with high levels of PHGDH.

In this line, to investigate the role of the PHGDH/HH axis in 5-FU resistance, we quantified the expression of GLI1, GLI2 and PTCH1 in HCT116 cells resistant to 5-FU (HCT116R), previously established in our lab [17]. Results showed an up-regulation of GLI1, GLI2 and PTCH1 expression (Fig. 6G), as well as HH target genes (Fig. 6H) in HCT116R cells respect to the sensitive ones, confirming the close link between the activation of HH signaling and resistance to 5-FU. In keeping, the combo treatment is effective in enhancing 5-FU sensitivity in HCT116R cells (Fig. 6I).

To further validate the correlation between HH signaling and 5-FU resistance, we analyzed RNA-seq data from The Cancer Genome Atlas (TCGA). We interrogated the open access expression and clinical databases TCGA-COAD and TCGA-READ specifically, focusing on patients who had received 5-FU-based chemotherapy. Based on clinical outcome following the treatment, patients were classified as R or NR (see methods). GSEA revealed significant upregulation of the HH signaling pathway in the NR group (NES = 1.509, p. adjusted = 0.0401), confirming the relevance of this pathway in sustaining resistance to 5-FU-based therapy (Fig. 6J). These findings from a large external dataset further corroborate our experimental results demonstrating the functional relationship between HH pathway activation and 5-FU resistance in CRC.

### JC19 administration boots 5-FU effects in a CRC xenograft mouse model

**Fig. 7:**
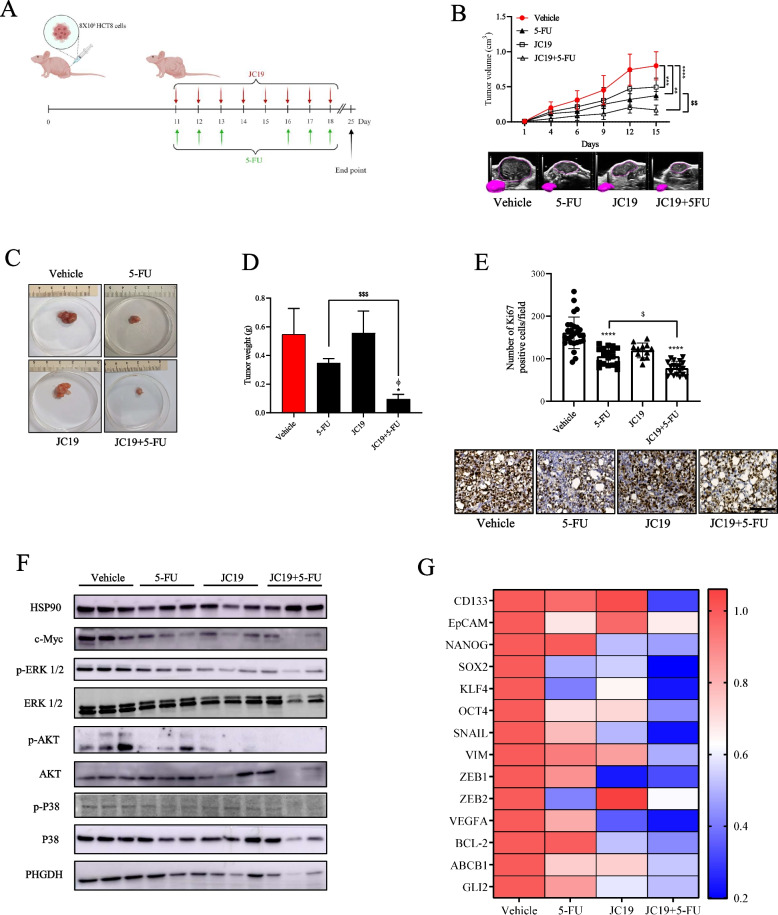
Inhibition of HH pathway boosts 5-FU toxic effect. **A** 8 × 10^6^ HCT8 cells were injected into the flanks of nu/nu mice. When tumors became palpable (day 11) mice were randomly divided in 4 groups (*n* = 6) and treated for 2 weeks as reported: Vehicle, 5-FU (25 mg/kg), JC19 (15 mg/kg) and a combination of both. **B** Tumor growth curve in mice treated as described in A (upper panel). Day 1 represents treatment start; analysis of tumor volume by Vevo LAZR-X photoacoustic imaging (lower panel) at the end point of the in vivo experiment. Three-dimensional rendering of ultrasound images of representative subcutaneous tumor masses is shown in the insert. t-test; ** *p* < 0.01, *** *p* < 0.001, **** *p* < 0.0001; $$ *p* < 0.001. **C** Representative images of dissected tumor samples. **D** Tumor weight of explanted tumors. t-test; * *p* < 0.05; $$$ *p* < 0.001. ϕ indicates synergistic effect (Bliss test > 0) with respect to single treatments. **E** Ki67 positive cells by immunostaining assay in tumor slices from mice treated as in A. Representative staining of Ki67 and H&E is shown below the bargraph (magnification 40 ×, scale bar: 50 μm); t-test; **** *p* < 0.0001; $ *p* < 0.05 **F** Immunoblot of proteins involved in proliferation and survival in explanted tumors. HSP90 immunoblot was performed to ensure equal loading. **G** Heatmap plot of differentially expressed genes in tumor tissues from vehicle, 5-FU, JC19 and combo treated mice. Vehicle group was used as comparator. Gene expression levels are expressed in color code from blue (low) to red (high) according to the color key scale bar (*n* = 3 per group)

Finally, we validated these results in an in vivo CRC xenograft model obtained by subcutaneously injecting HCT8 cells into the flanks of Fox1 nu/nu mice. Once tumors were palpable, mice were randomized and treated for two weeks with 5-FU or JC19 as monotherapy as already described [20] or with a combination of the two treatments as reported in the scheme in Fig. 7A. As shown by tumor growth curve, ultrasound images, and tumor weight of excised tumors at the experiment endpoint, combining JC19 with 5-FU is more effective in impairing xenograft growth than the 5-FU treatment (Fig. 7B-D). In agreement with these results, IHC staining revealed a significant reduction of Ki67-positive cells in tumor section from combo-treated mice compared to those receiving single-drug treatments (Fig. 7E). Finally, we confirmed the efficacy of the combo treatment by testing the activation/expression of proteins involved in proliferation and survival by western blotting analysis (Fig. 7F). In particular, we observed a lower activation of key proteins such as phospho ERK 1/2, phospho P38 and c-Myc combining 5-FU with JC19. Similarly, qPCR analysis conducted on tumor tissues highlights a strong downregulation of genes involved in invasion (i.e. SNAIL, VIM, ZEB1, ZEB2), cancer stem cells (CD133, EpCAM, NANOG, SOX2, KLF4, OCT4) and HH pathway (ABCB1, GLI2, BCL2, VEGF-A) in the combo group compared to single drug treatments (Fig. 7G). Interestingly, we confirmed that also in tumor tissues from mice treated with JC19 or with the combo, a significant decrease in PHGDH expression is revealed (Fig. 7F).

Overall, our results demonstrate that combining 5-FU with inhibition of HH signaling synergistically decreases CRC xenografts growth in nude mice.

## Discussion

Tumor cells undergo profound changes in their metabolic pathways to support rapid proliferation and survival in challenging environments [47]. However, some metabolic enzymes usually involved in energy metabolism or biosynthesis have proven to be involved in other “non-metabolic” crucial cellular functions, including regulating tumor growth, aggressiveness, and resistance to treatments [48]. Among these, PHGDH, traditionally characterized as a key enzyme in Ser metabolism regulation [1], has recently been implicated in non-metabolic functions. Actually, the dysregulation of Ser metabolism can enhance tumor aggressiveness and resistance to therapies by supporting cancer cells in stress-induced damage evasion and microenvironment adaptations [49]. Coherently many tumors upregulate the SSP mostly through PHGDH overexpression [50] and PHGDH has been associated with poor prognosis in several types of cancers [27, 51]. Notably, we recently demonstrated that PHGDH expression correlates with increased 5-FU resistance and that Ser availability is essential to sustain DNA damage response upon drug exposure in CRC [17]. These results are in line with previous studies underlying the importance of PHGDH activity and Ser availability in sustaining chemoresistance against different drugs by mediating glutathione biosynthesis and antioxidant responses. In particular, the high-PHGDH-mediated activation of SSP supports sorafenib resistance in hepatocellular carcinoma by sustaining enhanced antioxidant response [52]; similarly, PHGDH levels are significantly increased in lung adenocarcinoma that acquires resistance to erlotinib, regulating cellular redox balance and supporting adaptive response to counteract drug treatment [53]. In this line, increased Ser synthesis is associated with bortezomib resistance in multiple myeloma [54] and BRAF inhibitor resistance in different types of cancers [55]. Moreover, in triple negative breast cancer, PHGDH supports Ser synthesis functional to produce glutathione, which plays a protective role against doxorubicin-induced oxidative stress [56]. Finally, our group demonstrated that increased SSP supports purine biosynthesis and potentiates DNA damage response in 5-FU resistant CRC cells [17].

Besides these metabolic roles, PHGDH is emerging as a driver of cancer aggressiveness by modulating different mechanisms [12]. This led us to investigate novel non canonical roles of PHGDH in sustaining drug resistance. Transcriptomic analysis on PHGDH-silenced CRC cells highlighted, among others, a decrease in the activation of the HH pathway associated with the downregulation of PHGDH. This evidenced emerged as particularly relevant considering that it is known that the HH pathway supports the maintenance and survival of CSCs and hence is crucial to sustaining chemotherapy resistance to different drugs, such as platinum [57], 5-FU [45], paclitaxel [58], and doxorubicin [59].

Coherently, it has been demonstrated that high levels of GLI1, the main effector of HH signaling, are associated to higher incidence of tumor relapse in 5-FU-treated CRC patients [44]. Moreover, the inhibition of the HH signaling pathway is effective in reversing drug resistance in CRC cell lines [42, 60]. Indeed, the combination treatment of GANT61, an inhibitor of HH pathway, with 5-FU is more effective in decreasing cell viability and inhibiting the expression of stem cell markers in tumor organoids compared the sole anti-cancer drug treatment [61]. In this context, for the first time, we highlighted that the activation of the HH pathway depends on PHGDH. It is also worth of notice that, recently, a similar relationship between PHGDH and another key stemness-related pathway, namely the Wnt pathway, has been underlined [62], suggesting that PHGDH could influence stemness via non enzymatic function.

Notably, we also identified that this mechanism is mediated by PHGDH nuclear localization, further confirming the contribution of non-metabolic functions of this protein in mediating CRC aggressiveness. At nuclear level, PHGDH can associate with c-Myc, a transcription factor known to promote GLI1 expression interacting with the 5’-regulatory region of the *Gli1* gene [63], opening a new scenario on a possible role of PHGDH as transcriptional regulator.

In addition to this recently identified distinct subcellular localization, PHGDH is known to exhibit pronounced dynamic heterogeneity in its expression over tumor progression.

Differential expression in resistance-driving metabolic genes is known to explain the heterogeneity in drug response among tumor patients. Confirming previous evidence, heterogeneous PHGDH expression reflects a significant variability in PHGDH levels in 3D organoids and tumor samples derived from CRC patients. Of note we demonstrated that high PHGDH expression correlates with a worse prognosis and decreased responsiveness to 5-FU-based treatments.

Collectively, we demonstrated that besides its known role in increasing Ser availability, PHGDH sustains 5-FU resistance also through the up-regulation of HH signaling. In line, the combination of 5-FU with drugs inhibiting HH pathway acts synergistically to reduce cell viability of both human CRC patient-derived organoids and CRC xenografts in nude mice. These findings suggest that this combinatorial therapeutic strategy could be a promising approach for treating CRC.

## Conclusion

In conclusion, the data collected here pave the way to proposing PHGDH as a potential biomarker to predict the response to 5-FU-based chemotherapy treatments. This opens the development of targeted therapeutic approaches, where evaluating PHGDH levels could be useful to personalize treatments, thereby optimizing therapeutic efficacy and minimizing side effects.

## Supplementary Information

Supplementary Material 1: Supplementary Figure 1. PHGDH expression level correlates with CRC patient relapses. A) PHGDH protein expression in representative CRC human tissues. #CRC5, #CRC16, #CRC22, #CRC33: relapsing patients; #CRC1, #CRC8,#CRC26: non relapsing patients.

Supplementary Material 2: Supplementary Figure 2. PHGDH level supports CRC aggressiveness traits acquisition. A) PHGDH protein levels in PHGDH-silenced LS174T cells. Vinculin immunoblot was performed to ensure equal loading. Bargraph reports quantification of PHGDH level compared to vinculin and show the mean ± SEM of three independent experiments; t-test ** *p*<0.01, B) Survival fraction of LS174T cells transfected with shCTRL or shPHGDH after 48h of 5-FU treatment. Data are reported as mean ± SEM from three independent experiments; t-test **p<0.01, **** *p*<0.0001. C) γH2AX levels in transfected and parental LS174T cells. Vinculin and H2AX were used as loading control. D) Invasive abilities of LS174T cells transfected with shCTRL or shPHGDH. Cells were seeded in the upper compartment of 8 μm Transwell system coated with Matrigel. After 16h invaded cells were stained with Crystal Violet and counted. Representative images of invaded CRC cells are shown below the bargraphs (magnification 20×, scale bar: 100 μm). Data are reported as mean ± SEM from three independent experiments; t-test **** *p*<0.0001.

Supplementary Material 3: Supplementary Figure 3 High PHGDH levels promote the HH pathway. A) PTCH1, GLI1 and GLI2 mRNA expression levels in LS174T and RKO cells analyzed by qPCR. High-PHGDH expressing cells were used as comparator. Data are reported as mean ± SEM from three independent experiments; t-test ** *p*<0.01, *** *p*<0.001. B) Representative images of western blot for PHGDH and β-Actin expression in LS174T and RKO cells. C) Survival fraction of RKO, RKO OE PHGDH, HCT8 and HCT8 shPHGDH cells treated or not with 5 μM 5-FU in combination with 10 μM GANT61 for 48h. Data are reported as mean ± SEM from three independent experiments; ANOVA; **** *p*<0.0001.

Supplementary Material 4.

Supplementary Material 5.

Supplementary Material 6.

## Data Availability

RNA-seq data are publicly available through the GEO repository (accession number: GSE294639), including both raw FASTQ files and Salmon-derived raw count quant.sf files.

## Abbreviations

CRC: Colorectal cancer
CSC: Cancer stem cell
RFS: Relapse-free survival
EMT: Epithelial-mesenchymal transition
5-FU: 5-Fluorouracil
GSEA: Gene Set Enrichment Analysis
HH: Hedgehog
IHC: Immunohistochemistry
NR: Non-responder
OS: Overall survival
PDCO: Patient-derived cancer organoid
PHGDH: Phosphoglycerate dehydrogenase
R: Responder
Ser: Serine
Ser/Gly: Serine and Glycine
SSP: Serine synthesis pathway
