# Supplementary figures and images for: PHGDH drives 5-FU chemoresistance in colorectal cancer through the Hedgehog signaling
Source: J Exp Clin Cancer Res. 2025 Jul 10;44:198. doi: 10.1186/s13046-025-03447-y (PMC12243184; doi:10.1186/s13046-025-03447-y)

Suppl figure 1

A

## Tumor Tissues

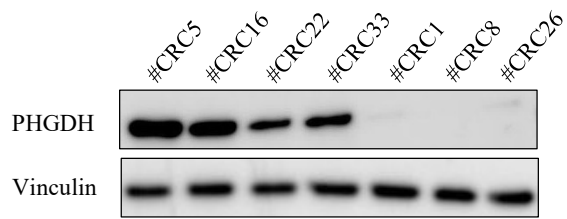

Supplement: Supplementary file 1 — Supplementary Material 1: Supplementary Figure 1. PHGDH expression level correlates with CRC patient relapses. A) PHGDH protein expression in representative CRC human tissues. #CRC5, #CRC16, #CRC22, #CRC33: relapsing patients; #CRC1, #CRC8,#CRC26: non relapsing patients. [file 13046_2025_3447_MOESM1_ESM.pdf]

Suppl figure 2

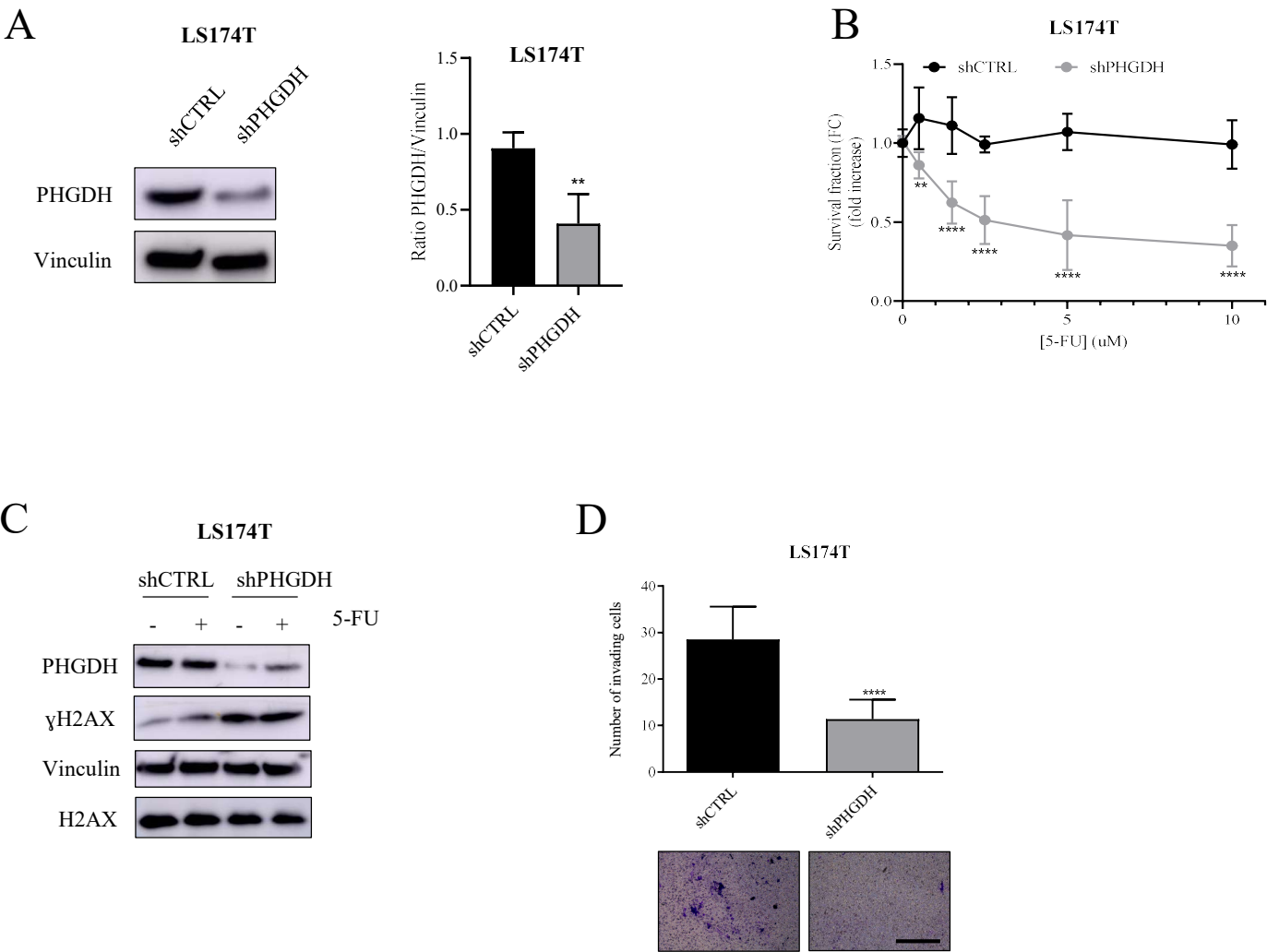

Supplement: Supplementary file 2 — Supplementary Material 2: Supplementary Figure 2. PHGDH level supports CRC aggressiveness traits acquisition. A) PHGDH protein levels in PHGDH-silenced LS174T cells. Vinculin immunoblot was performed to ensure equal loading. Bargraph reports quantification of PHGDH level compared to vinculin and show the mean ± SEM of three independent experiments; t-test ** p<0.01, B) Survival fraction of LS174T cells transfected with shCTRL or shPHGDH after 48h of 5-FU treatment. Data are reported as mean ± SEM from three independent experiments; t-test **p<0.01, **** p<0.0001. C) γH2AX levels in transfected and parental LS174T cells. Vinculin and H2AX were used as loading control. D) Invasive abilities of LS174T cells transfected with shCTRL or shPHGDH. Cells were seeded in the upper compartment of 8 μm Transwell system coated with Matrigel. After 16h invaded cells were stained with Crystal Violet and counted. Representative images of invaded CRC cells are shown below the bargraphs (magnification 20×, scale bar: 100 μm). Data are reported as mean ± SEM from three independent experiments; t-test **** p<0.0001. [file 13046_2025_3447_MOESM2_ESM.pdf]

Suppl figure 3

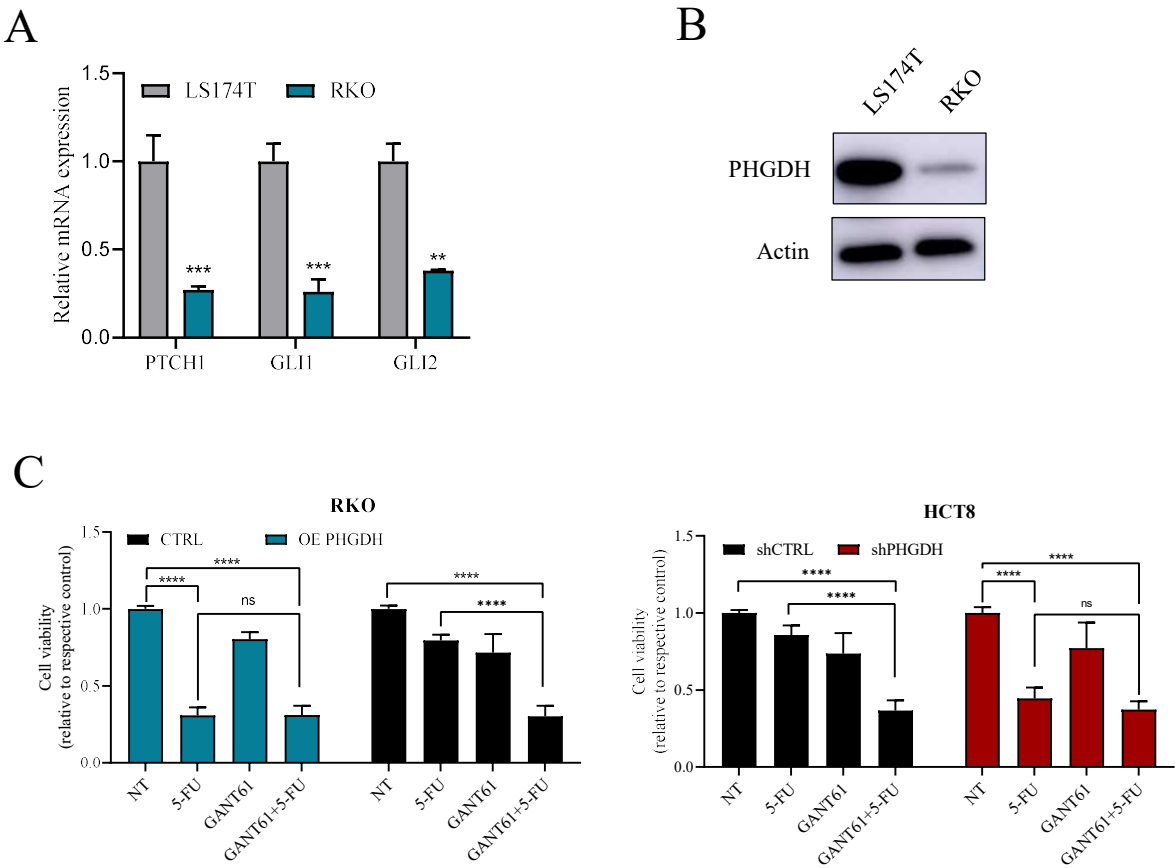

Supplement: Supplementary file 3 — Supplementary Material 3: Supplementary Figure 3 High PHGDH levels promote the HH pathway. A) PTCH1, GLI1 and GLI2 mRNA expression levels in LS174T and RKO cells analyzed by qPCR. High-PHGDH expressing cells were used as comparator. Data are reported as mean ± SEM from three independent experiments; t-test ** p<0.01, *** p<0.001. B) Representative images of western blot for PHGDH and β-Actin expression in LS174T and RKO cells. C) Survival fraction of RKO, RKO OE PHGDH, HCT8 and HCT8 shPHGDH cells treated or not with 5 μM 5-FU in combination with 10 μM GANT61 for 48h. Data are reported as mean ± SEM from three independent experiments; ANOVA; **** p<0.0001. [file 13046_2025_3447_MOESM3_ESM.pdf]
