# Supplementary material for: PHGDH drives 5-FU chemoresistance in colorectal cancer through the Hedgehog signaling
Source: J Exp Clin Cancer Res. 2025 Jul 10;44:198. doi: 10.1186/s13046-025-03447-y (PMC12243184; doi:10.1186/s13046-025-03447-y)
Supplement: Supplementary file 4 — Supplementary Material 4. [file 13046_2025_3447_MOESM4_ESM.docx]

| **Gene** | **Sequence** |
| --- | --- |
| PHGDH-FW | AGGCTCGCATCAGTGTCC |
| PHGDH-REV | ATCTCTCACGGGGGTTGTG |
| EpCAM-FW | TGTGGTGATAGCAGTTGTTGC |
| EpCAM-REV | CTATGCATCTCACCCATCTCC |
| LGR5-FW | CTTCCAACCTCAGCGTCTTC |
| LGR5-REV | TTTCCCGCAAGACGTAACTC |
| CD133-FW | GCTTCAGGAGTTTCATGTTGG |
| CD133-REV | GGGGAATGCCTACATCTGG |
| VEGF-FW | CACTGAGGAGTCCAACATCAC |
| VEGF-REV | AGGAAGCTCATCTCTCCTATGT |
| ALDH1-FW | GCACGCCAGACTTACCTGTC |
| ALDH1-REV | CCACTCACTGAATCATGCCA |
| ABCB1-FW | AGTGAAAAGGTTGTCCAAG |
| ABCB1-REV | AGTCTGCATTCTGGATGG |
| KLF4-FW | AGACAGTCTGTTATGCACTGTGG |
| KLF4-REV | TGTTCTGCTTAAGGCATACTTGG |
| CMYC-FW | CGGAACTCTTGTGCGTAAGG |
| CMYC-REV | ACTCAGCCAAGGTTGTGAGG |
| ECAD-FW | AGGCCAAGCAGCAGTACATT |
| ECAD-REV | ATTCACATCCAGCACATCCA |
| KFL4-FW | AGACAGTCTGTTATGCACTGTGG |
| KLF4-REV | TGTTCTGCTTAAGGCATACTTGG |
| NCAD-FW | CCTCCAGAGTTTACTGCCATGAC |
| NCAD-REV | GTAGGATCTCCGCCACTGATTC |
| BCL2-FW | ATCGCCCTGTGGATGACTGAGT |
| BCL2-REV | GCCAGGAGAAATCAAACAGAGGC |
| GLI1-FW | CCCAGTACATGCTGGTGGTT |
| GLI1-REV | GCTTTACTGCAGCCCTCGT |
| GLI2-FW | CTCAGCCCCGCTGATGTGGC |
| GLI2-REV | TCAGCAGGTCCCCGTAGGGC |
| PATCH1-FW | GGCAGCGGTAGTAGTGGTGTTC |
| PATCH1-REV | TGTAGCGGGTATTGTCGTGTGTG |
| B2M-FW | AGTATGCCTGCCGTGTGAAC |
| B2M-REV | GCGGCATCTTCAAACCTCCA |

**Supplementary Table 1**
