# Supplementary material for: PHGDH drives 5-FU chemoresistance in colorectal cancer through the Hedgehog signaling
Source: J Exp Clin Cancer Res. 2025 Jul 10;44:198. doi: 10.1186/s13046-025-03447-y (PMC12243184; doi:10.1186/s13046-025-03447-y)
Supplement: Supplementary file 5 — Supplementary Material 5. [file 13046_2025_3447_MOESM5_ESM.pdf]

| Patient n° | Histological diagnosis | Grade | pTMN stage | Relapse (YES=1; NO=0) |
|---|---|---|---|---|
| #1 | Intestinal mucinous adenocarcinoma | G1 | pT3N1a | 0 |
| #3 | Invasive intestinal adenocarcinoma NOS, moderately differentiated | G1 | N/A | 0 |
| #4 | Adenocarcinoma NOS with colloid features | G1 | pT3N1c | 0 |
| #5 | Invasive intestinal adenocarcinoma NOS | G1 | pT2N1b | 1 |
| #6 | Invasive intestinal adenocarcinoma NOS | G1 | pT3N1b | N/A |
| #7 | Invasive intestinal adenocarcinoma NOS | G1 | pT3N0 | N/A |
| #8 | Invasive intestinal adenocarcinoma NOS with micropapillary (20%) and colloid (20%) features | G2 | pT3N2b | 0 |
| #9 | Adenocarcinoma NOS, well differentiated | G1 | pT3N1b | 1 |
| #10 | Adenocarcinoma NOS | G2 | pT3N0 | 0 |
| #11 | Intestinal adenocarcinoma NOS | G2 | pT3N1b | N/A |
| #12 | Adenocarcinoma NOS with mucinous features (25%) | G2 | pT3N0 | 0 |
| #13 | Adenocarcinoma | N/A | N/A | 0 |
| #14 | Adenocarcinoma NOS | G1 | pT2N0 | N/A |
| #15 | Adenocarcinoma NOS | G1 | pT3N0 | N/A |
| #16 | Intestinal adenocarcinoma with colloid features, poorly differentiated | G3 | pT4N1aM1 | 1 |
| #17 | Adenocarcinoma NOS | G1 | pT3N0 | 0 |
| #18 | Adenocarcinoma | N/A | N/A | 0 |
| #19 | Adenocarcinoma | N/A | N/A | N/A |
| #20 | Intestinal adenocarcinoma NOS | G2-G3 | pT3N0 | N/A |
| #21 | Adenocarcinoma intestinale invasivo, NOS | G1 | pT2N0 | N/A |
| #22 | Adenocarcinoma NOS | G3 | pT3N1b | 1 |
| #23 | Adenocarcinoma NOS with colloid features (40%) | G1 | pT2N1a | 0 |
| #24 | Adenocarcinoma NOS | G1 | pT1N0 | N/A |
| #25 | Intestinal adenocarcinoma NOS | G2 | pT2N0 | N/A |
| #26 | Adenocarcinoma NOS | G3 | pT4aN1b | 0 |
| #27 | Adenocarcinoma | N/A | N/A | N/A |
| #33 | Adenocarcinoma NOS | G1 | pT3N0 | 1 |

**Table 2:** Clinicopathological characteristics of CRC patients.
