# Supplementary material for: PHGDH drives 5-FU chemoresistance in colorectal cancer through the Hedgehog signaling
Source: J Exp Clin Cancer Res. 2025 Jul 10;44:198. doi: 10.1186/s13046-025-03447-y (PMC12243184; doi:10.1186/s13046-025-03447-y)
Supplement: Supplementary file 6 — Supplementary Material 6. [file 13046_2025_3447_MOESM6_ESM.docx]

| **Patient** | **Age at surgery** | **Sex** | **Tumor location** | **Surgery** | **Histological diagnosis** | **Grade of differentiation** | **pTNM stage** | **PHGDH score** | **Adjuvant therapy** | **Response/Non-response** |
| --- | --- | --- | --- | --- | --- | --- | --- | --- | --- | --- |
| A1 | 78 | F | Sigmoid colon | Anterior rectal resection | Adenocarcinoma NOS | G2 | T3N0Mx | 160 | 5-Fluorouracil | R |
| A2 | 73 | F | Sigmoid colon | Anterior rectal resection | Adenocarcinoma NOS | G2 | T3N0Mx | 0 | 5-Fluorouracil | NR |
| A3 | 64 | F | Transverse colon | Transversal resection | Adenocarcinoma NOS | G2 | T3N2bM1a | 80 | 5-Fluorouracil | R |
| A4 | 64 | M | Rectum | Anterior rectal resection | Adenocarcinoma NOS | G2 | T3N2M1a | 0 | 5-Fluorouracil | NR |
| A5 | 61 | M | Right flexure | Right hemicolectomy | Adenocarcinoma NOS | G2 | T2N1aMx | 5 | 5-Fluorouracil | R |
| A6 | 68 | M | Sigmoid colon | Anterior rectal resection | Adenocarcinoma NOS | G2 | T3N1b M1a | 160 | 5-Fluorouracil | NR |
| A7 | 61 | F | Sigmoid colon | Anterior rectal resection | Adenocarcinoma with colloid features | G2 | T3N2aMx | 10 | 5-Fluorouracil | NR |
| A8 | 62 | F | Rectum | Anterior rectal resection | Adenocarcinoma NOS | G2 | T3N2aM1a | 0 | 5-Fluorouracil | NR |
| A9 | 61 | F | Recto-sigmoid junction | Anterior rectal resection | Adenocarcinoma NOS | G1 | T3N1aMx | 160 | 5-Fluorouracil | NR |
| A10 | 72 | F | Recto-sigmoid junction | Anterior rectal resection | Adenocarcinoma with colloid features | G2 | T3N1cMx | 10 | 5-Fluorouracil | R |
| A11 | 71 | M | Recto-sigmoid junction | Anterior rectal resection | Adenocarcinoma NOS | G2 | T2N1aMx | 0 | 5-Fluorouracil | R |
| A12 | 74 | M | Cecum | Right hemicolectomy | Adenocarcinoma NOS | G2 | T3N1aMx | 30 | 5-Fluorouracil | R |
| A13 | 75 | M | Signoid Colon | Anterior rectal resection | Adenocarcinoma NOS | G2 | T2N1aM1a | 0 | 5-Fluorouracil | NR |
| A14 | 70 | F | Rectum | Anterior rectal resection | Adenocarcinoma NOS | G2 | T2N1bMx | N/A | 5-Fluorouracil | R |
| A15 | 64 | M | Rectum | Anterior rectal resection | Adenocarcinoma NOS | G2 | T3N0M1a | 70 | 5-Fluorouracil | R |
| A16 | 43 | F | Rectum | Anterior rectal resection | Adenocarcinoma NOS | G2 | T3N1bMx | 60 | 5-Fluorouracil | R |
| A17 | 63 | M | Recto-sigmoid junction | Anterior rectal resection | Adenocarcinoma NOS | G2 | T3N1cMx | 15 | 5-Fluorouracil | R |
| A18 | 46 | F | Recto-sigmoid junction | Anterior rectal resection | Adenocarcinoma NOS | G2 | T3N1cMx | 120 | 5-Fluorouracil | R |
| A19 | 75 | M | Ascending colon | Right hemicolectomy | Adenocarcinoma NOS | G2 | T3N1aMx | 0 | 5-Fluorouracil | R |
| A20 | 70 | M | Descending colon | Left hemicolectomy | Adenocarcinoma NOS | G2 | T2N1aM1a | 10 | 5-Fluorouracil | NR |
| A21 | 68 | M | Descending colon | Left hemicolectomy | Adenocarcinoma NOS | G2 | T3N2aMx | 150 | 5-Fluorouracil | NR |
| A22 | 52 | F | Rectum | Anterior rectal resection | Adenocarcinoma NOS | G2 | T0N1aMx | N/A | 5-Fluorouracil | NR |
| A23 | 77 | M | Descending colon | Left hemicolectomy | Adenocarcinoma NOS | G2 | T3N1aMx | 140 | 5-Fluorouracil | R |
| A24 | 79 | F | Descending colon | Left hemicolectomy | Adenocarcinoma NOS | G2 | T3N1aMx | 80 | 5-Fluorouracil | R |
| A25 | 81 | F | Cecum | Right hemicolectomy | Adenocarcinoma NOS | G2 | T3N1bMx | 0 | 5-Fluorouracil | NR |
| A26 | 45 | F | Rectum | Anterior rectal resection | Adenocarcinoma NOS | G2 | T3N2aM1a | 0 | 5-Fluorouracil | R |
| A27 | 55 | M | Descending colon | Left hemicolectomy | Adenocarcinoma NOS | G2 | T3N2aM1a | 0 | 5-Fluorouracil | NR |
| A28 | 66 | F | Rectum | Anterior rectal resection | Adenocarcinoma NOS | G2 | T3N0Mx | N/A | 5-Fluorouracil | NR |
| A29 | 72 | F | N/A | N/A | Adenocarcinoma NOS | G2 | T2N1cM1a | 40 | 5-Fluorouracil | NR |
| A30 | 72 | M | Left flexure | Left hemicolectomy | Adenocarcinoma NOS | G2 | T3N1aM1a | 15 | 5-Fluorouracil | NR |
| A31 | 59 | F | Ascending colon | Right hemicolctomy | Adenocarcinoma NOS | G2 | T3N1aMx | 180 | 5-Fluorouracil | NR |
| A32 | 64 | F | Descending colon | Anterior rectal resection | Adenocarcinoma NOS | G2 | T3N1bMx | 20 | 5-Fluorouracil | R |
| A33 | 63 | M | Descending colon | Left hemicolectomy | Adenocarcinoma NOS | G2 | T3N1bMx | 60 | 5-Fluorouracil | NR |
| A34 | 68 | F | Cecum | Right hemicolectomy | Adenocarcinoma NOS | G2 | T3N1bM1a | 40 | 5-Fluorouracil | NR |
| A35 | 48 | F | Ascending colon | Right hemicolectomy | Adenocarcinoma NOS | G2 | T3N0Mx | 30 | 5-Fluorouracil | R |
| A36 | 80 | M | Rectum | Anterior rectal resection | Adenocarcinoma NOS | G2 | T3N2aMx | 270 | 5-Fluorouracil | NR |
| A37 | 69 | F | Sigmoid colon | Anterior rectal resection | Adenocarcinoma NOS | G2 | T3N2bMx | 30 | 5-Fluorouracil | NR |
| A38 | 75 | M | Sigmoid colon | Anterior rectal resection | Adenocarcinoma NOS | G2 | T2N1aMx | 120 | 5-Fluorouracil | R |
| A39 | 53 | F | Ascending colon | Right hemicolectomy | Adenocarcinoma NOS | G2 | T3N1bMx | 240 | 5-Fluorouracil | NR |
| A40 | 66 | F | Descending colon | Left hemicolectomy | Mucinous adenocarcinoma | G3 | T3N2bMx | 140 | 5-Fluorouracil | NR |
| A41 | 67 | M | Sigmoid colon | Anterior rectal resection | Adenocarcinoma NOS | G2 | T3N1cMx | 120 | 5-Fluorouracil | NR |
| A42 | 68 | M | Rectum | Anterior rectal resection | Adenocarcinoma NOS | G2 | T3N2aMx | 5 | 5-Fluorouracil | R |
| A43 | 68 | M | Transverse colon | Transverse resection | Adenocarcinoma NOS | 2 | T3N2aMx | 0 | 5-Fluorouracil | R |
| A44 | 57 | M | Rectum | Anterior rectal resection | Adenocarcinoma NOS | 3 | T4bN1bMx | 5 | 5-Fluorouracil | NR |
| A45 | 76 | M | Rectum | Anterior rectal resection | Adenocarcinoma NOS | 2 | T3N2aMx | 100 | 5-Fluorouracil | R |
| A46 | 62 | F | Sigmoid colon | Anterior rectal resection | Adenocarcinoma NOS | 2 | T3N0M1a | 240 | 5-Fluorouracil | R |
| A47 | 60 | M | Rectum | Anterior rectal resection | Adenocarcinoma NOS | 2 | T2N1aMx | 0 | 5-Fluorouracil | R |
| A48 | 48 | F | Descending colon | Left hemicolectomy | Adenocarcinoma NOS | 2 | T3N1bMx | 120 | 5-Fluorouracil | R |
| A49 | 62 | M | Sigmoid colon | Anterior rectal resection | Adenocarcinoma NOS | 2 | T3N1aMx | 25 | 5-Fluorouracil | R |
| A50 | 81 | M | Sigmoid colon | Anterior rectal resection | Adenocarcinoma NOS | 2 | T3N1bMx | 160 | 5-Fluorouracil | NR |
| A51 | 70 | F | Sigmoid colon | Anterior rectal resection | Adenocarcinoma NOS | 2 | T3N1aMx | 160 | 5-Fluorouracil | R |
| A52 | 66 | M | Sigmoid colon | Anterior rectal resection | Adenocarcinoma NOS | 2 | T2N1aMx | 10 | 5-Fluorouracil | R |
| A53 | 58 | F | Rectum | Anterior rectal resection | Adenocarcinoma NOS | 2 | T3N1aMx | 0 | 5-Fluorouracil | R |

**Supplementary Table 3:** Clinicopathological characteristics of CRC patients and relationship between PHGDH expression and response to therapy. R: Response; NR: Non-response
